# Baseline Toxicity Model to Identify the Specific and Nonspecific Effects of Per- and Polyfluoroalkyl Substances in Cell-Based Bioassays

**DOI:** 10.1021/acs.est.3c09950

**Published:** 2024-02-23

**Authors:** Weiping Qin, Luise Henneberger, Juliane Glüge, Maria König, Beate I. Escher

**Affiliations:** † Department of Cell Toxicology, UFZ−Helmholtz Centre for Environmental Research, Leipzig 04318, Germany; ‡ Environmental Toxicology, Department of Geosciences, Eberhard Karls University Tübingen, Schnarrenbergstr. 94-96, Tübingen DE-72076, Germany; § Institute of Biogeochemistry and Pollutant Dynamics, ETH Zürich, Zürich 8092, Switzerland

**Keywords:** PFAS, membrane concentration, liposome-water distribution ratio, potential adverse outcome pathways, peroxisome proliferator-activated receptors

## Abstract

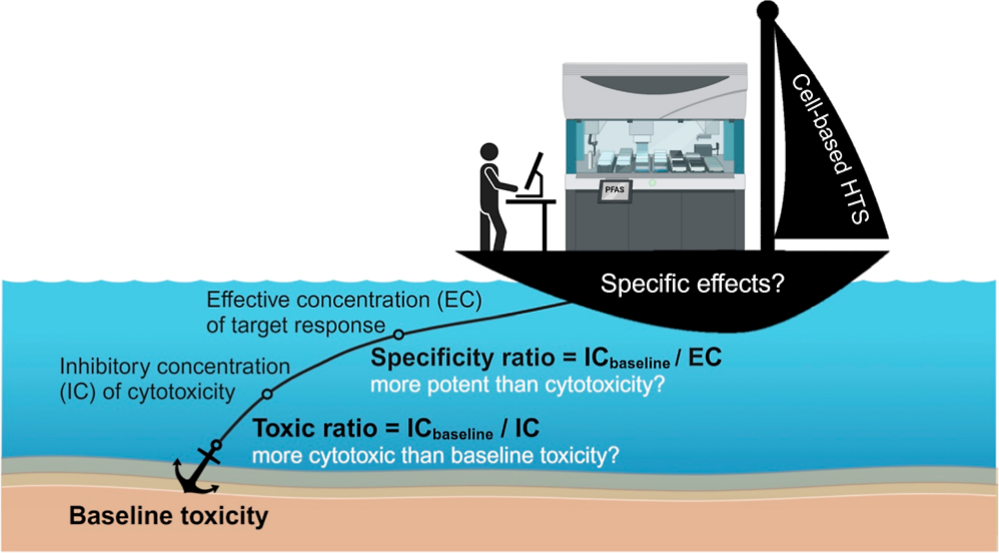

High-throughput screening is a strategy to identify potential adverse outcome pathways (AOP) for thousands of per- and polyfluoroalkyl substances (PFAS) if the specific effects can be distinguished from nonspecific effects. We hypothesize that baseline toxicity may serve as a reference to determine the specificity of the cell responses. Baseline toxicity is the minimum (cyto)toxicity caused by the accumulation of chemicals in cell membranes, which disturbs their structure and function. A mass balance model linking the critical membrane concentration for baseline toxicity to nominal (i.e., dosed) concentrations of PFAS in cell-based bioassays yielded separate baseline toxicity prediction models for anionic and neutral PFAS, which were based on liposome-water distribution ratios as the sole model descriptors. The specificity of cell responses to 30 PFAS on six target effects (activation of peroxisome proliferator-activated receptor (PPAR) gamma, aryl hydrocarbon receptor, oxidative stress response, and neurotoxicity in own experiments, and literature data for activation of several PPARs and the estrogen receptor) were assessed by comparing effective concentrations to predicted baseline toxic concentrations. HFPO–DA, HFPO–DA-AS, and PFMOAA showed high specificity on PPARs, which provides information on key events in AOPs relevant to PFAS. However, PFAS were of low specificity in the other experimentally evaluated assays and others from the literature. Even if PFAS are not highly specific for certain defined targets but disturb many toxicity pathways with low potency, such effects are toxicologically relevant, especially for hydrophobic PFAS and because PFAS are highly persistent and cause chronic effects. This implicates a heightened need for the risk assessment of PFAS mixtures because nonspecific effects behave concentration-additive in mixtures.

## Abstract 2

A baseline toxicity prediction model for cell-based bioassays demonstrates that PFAS act as baseline toxicants in many in vitro bioassays.

## Introduction

The threat that per- and polyfluoroalkyl substances (PFAS) pose to human health has been a great concern for society. The concern has expanded from perfluorooctanoic acid (PFOA) and perfluorooctanesulfonic acid (PFOS) to thousands of PFAS with diverse structures and unclear toxicological effects. The OECD broadened the definition of PFAS to at least one perfluorinated carbon,^1^ which implies that there are now more than 14,000 PFAS chemicals in the CompTox Chemistry Dashboard.^2^ Traditional methods can no longer cope with the risk assessment of such large numbers of PFAS. New approach methodologies (NAM), especially those based on high-throughput screening (HTS) with cellular assays, provide a strategy of extensive screening for molecular initiating events and key events in adverse outcome pathways (AOP) because cell responses can serve as early warning signals. PFAS may trigger cell responses through several cellular toxicity pathways, including reactive (oxidative stress),^3^ specific (receptor-mediated signaling pathways),^4^ and nonspecific toxicity.

Baseline toxicity, also known as narcosis, is a common, nonspecific effect caused by an accumulation of chemicals in cell membranes that interfere with membrane function and destroy membrane integrity.^5^ Chemicals that act as baseline toxicants can trigger specific effects at critical membrane concentrations. Baseline toxicity does not equate to low toxicity; very hydrophobic chemicals can still be very potent through baseline toxicity, i.e., act in low concentrations. Hence, baseline toxicity may serve as an anchor or reference state to determine the degree of specificity of PFAS in cell-based bioassays because specific effects occur at even lower concentrations.

**Figure 1:**
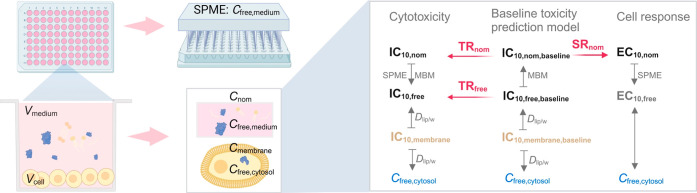
An overview of the dose-metrics and baseline toxicity prediction models for cell-based bioassays. Abbreviation: *V*_medium_ or*V*_cell_ = volume of medium or cells, *C*_nom_ = nominal concentration, *C*_free_ = free concentration, SPME = solid-phase microextraction, MBM = mass balance model, IC = inhibitory concentration, EC = effect concentration, *D*_lip/w_ = distribution ratio between liposomes and water, TR = toxic ratio, and SR = specificity ratio.

Baseline toxicity occurs when critical membrane burdens are exceeded. Escher et al.^6^ derived the critical membrane concentration of baseline toxicants that causes 10% of cytotoxicity IC_10,membrane,baseline_ as 69 mmol/L_lip_ from eight different cell lines. IC_10,membrane,baseline_ was independent of the cell line and constant for all organic chemicals. As in vitro bioassay responses are reported as nominal (i.e., dosed) concentrations in bioassay medium, one needs to link IC_10,membrane,baseline_ and freely dissolved concentrations (IC_10,free,baseline_) to nominal concentrations (IC_10,nom,baseline_), which can be accomplished by mass balance models (MBM).^7^ While IC_10,membrane,baseline_ is constant, IC_10,free,baseline_ is dependent on the partitioning of chemicals into membrane lipid bilayers, which can be simulated by phospholipid vesicles, so-called liposomes. The IC_10,free,baseline_ can vary over many orders of magnitude. Hydrophobic chemicals have much lower IC_10,free,baseline_ values than hydrophilic and charged chemicals. IC_10,nom,baseline_ is determined by the experimental conditions and binding affinities of PFAS to proteins and lipids in the medium and cells. An overview of these metrics can be found in Figure 1.

The inhibitory concentration at 10% cytotoxicity (IC_10,nom_) can be derived from the concentration–cytotoxicity curves using nominal concentrations in the bioassay. The ratio between IC_10,nom,baseline_ and experimental IC_10,nom_ is called the toxic ratio (TR_nom_).^8,9^ At lower, noncytotoxic concentrations, many in vitro bioassays trigger specific effects, such as the activation of receptors or adaptive stress responses. The effect concentration at 10% effect (EC_10,nom_) can be compared to the cytotoxicity IC_10,nom,baseline_ to derive the specificity ratio (SR_nom_).^9^ If TR_nom_ or SR_nom_ are close to 1, cell death or response may be caused by baseline toxicity. A positive artifact due to the so-called cytotoxicity burst may appear when cells are close to death. High TR_nom_ or SR_nom_ values suggest that chemicals specifically trigger cytotoxicity or defined targets at lower concentrations than baseline toxicity.

The specific effects refer to the signals from defined targets, which can be distinguished from the nonspecific baseline toxicity. Here are several cases among the numerous toxicological studies. Oxidative stress is a common mechanism of PFAS-induced cytotoxicity.^3^ For example, acute exposure to PFOS induced excessive production of reactive oxidative species, resulting in the apoptosis of mouse islet β-TC-6 cells^10^ and human neuroblastoma SH-SY5Y cells.^11^ As a cellular defense, PFOS was found to trigger the nuclear factor erythroid 2-related factor 2 (Nrf2)-antioxidant response element (ARE) pathway, which plays a critical role in cellular protection against toxicity and oxidative stress from chemical stressors.^12^ Besides, PFAS may disrupt endocrine homeostasis by interacting with receptor-mediated signaling pathways.^4^ For example, PFOS promoted adipogenesis via peroxisome proliferator-activated receptors (PPARs) in mouse preadipocyte 3T3-L1 cells;^13^ PFDA and PFDoA might disrupt the function of the thyroid hormone system via aryl hydrocarbon receptor (AhR) in rat pituitary GH3 cell;^14^ PFOS stimulated insulin secretion via membrane G-protein coupled receptor (GPR) 40 in mouse islet β-TC-6 cells.^15^ Neurotoxicity is also of concern; for example, PFOS enhanced nerve growth factor-induced neurite outgrowth in rat pheochromocytoma PC12 cells.^16^ To relate the observed effects to baseline toxicity is a way to allow comparison between different test systems and end points and to identify effects that are of particular concern due to high TR_nom_ or SR_nom_.

Tox21 and ToxCast are programs of multiple federal agencies, including the U.S. Environmental Protection Agency (EPA) and the National Toxicology Program,^17^ aiming at efficiently identifying potential AOPs for specified chemicals at molecular, cellular, and organ levels through HTS to facilitate risk assessments of chemicals. Currently, in vitro effect data for 160 PFAS from the EPA have been published.^18−21^ Transactivation assays encompassing 81 diverse transcription factors were screened with 142 PFAS to describe the activation of nuclear receptor-mediated signaling pathways.^18^ 148 biomarkers relevant to the immune system were measured with 147 PFAS to inform mechanisms of immunotoxicity.^19^ A NAM battery for developmental neurotoxicity was developed to evaluate the effects of 160 PFAS on neural network formation and function.^20^ Radioactive iodide uptake high-throughput assay was used to test 149 PFAS for potential thyroid disruption.^21^ However, data evaluation and ranking scores were done independently with diverse conclusions, which makes it difficult to determine which PFAS are of the highest concern and which in vitro end points are relevant for human health.

In the present study, we systematically assessed the role of PFAS distribution in bioassay systems and how this affected the different dose-metrics in bioassays (Figure 1). The free concentrations of 10 anionic and one partially charged PFAS in the bioassays run in a 96-well plate format were measured by solid-phase microextraction (SPME) to derive IC_10,free_ from a free concentration–cytotoxicity curve. Experimental IC_10,free_ values were compared with IC_10,free_ predicted by the MBM. A validated MBM was used to link the known critical membrane concentrations for baseline toxicity IC_10,membrane,baseline_ to IC_10,nom,baseline_.

Four cell-based HTS were selected as experimental batteries, including three reporter gene assays targeting oxidative stress (AREc32 assay), nuclear receptors of PPARγ and AhR, as well as an image-based neurotoxicity assay that quantifies inhibition of neurite outgrowth of differentiated SH-SYSY cells, because these targets may be relevant to PFAS according to previous studies.^11−14,16^ 24 PFAS were then tested in a 384-well plate format. The cytotoxicity IC_10,nom_ and the effect concentrations EC_10,nom_ from these assays were compared with the IC_10,nom,baseline_ to derive TR_nom_ and SR_nom_, which were used to determine if the effects of PFAS are specific. We also demonstrated the applicability of the approach to other assays by evaluating the bioassay responses of 16 PFAS from the literature for their degree of specificity.

## Theory

### Free Concentrations of PFAS in Bioassays

The nominal concentration (*C*_nom_) in a bioassay is the total amount of chemicals (*n*_tot_) dosed divided by the total volume of the bioassay system (*V*_tot_), which is composed of medium (*V*_medium_) and cells (*V*_cell_) .
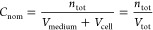
1

Fetal bovine serum (FBS) in medium serves as a reservoir of reversibly bound chemical and also facilitates a faster equilibrium of chemical between medium and cells,^22^ and thus, the free concentration of PFAS in medium (*C*_free,medium_) is usually lower than *C*_nom_. An MBM that accounts for the binding of chemicals to proteins and lipids in the medium and cells (eq 2) has been developed and validated experimentally for chemicals.^23^
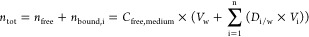
2

*C*_free,medium_ is related to the *C*_nom_ by inserting eq 1 in 2 yielding eq 3 with distribution ratios between the medium and water (*D*_medium/w_) and between the cells and water (*D*_cell/w_), as well as volumes of protein and lipid (*V*_protein+lipid_) in cells and medium. The detailed derivation of eq 3 is in Supporting Information Text S1.
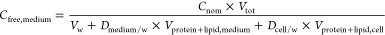
3

*D*_medium/w_ and *D*_cell/w_ can be measured experimentally or predicted by MBMs with the assumption that proteins and lipids are the main sorption phases in the medium and cells. Bovine serum albumin (BSA) serves as a surrogate for protein binding in medium (*D*_BSA/w_) and liposomes for partitioning to membrane lipids (*D*_lip/w_). Then, *D*_medium/w_ can be predicted by eq 4.
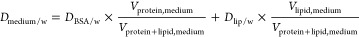
4

The most abundant proteins in cells are structural proteins (SP), for which muscle proteins are better surrogates than BSA.^24^ Analogously, *D*_cell/w_ can be predicted by eq 5 with *D*_SP/w_ referring to the distribution ratio between SP and water.
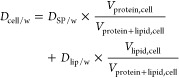
5

Note that in the previous baseline toxicity model,^7^ we had defined the *D*_medium/w_ and *D*_cell/w_ by including the water, protein, and lipid volumes in medium and cells, but here only protein and lipid volumes were defined as the sorption phases. *C*_free,medium_ can be calculated from *C*_nom_ by inserting eqs 4 and 5 in eq 3.
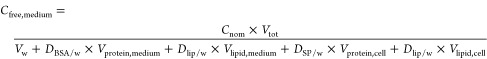
6

### Membrane and Free Concentrations Related to Baseline Toxicity

Chemicals inevitably accumulate in the membrane during the cellular uptake from the medium into the cytosol. The partitioning to membrane lipid bilayers can be simulated by phospholipid vesicles, so-called liposomes. The distribution ratio (*D*_lip/w_ (pH = 7.4)) is the ratio of the concentration of a chemical bound to liposomes (*C*_lip_) divided by the free concentration in the water phase (*C*_w_).
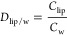
7

Some ionized PFAS may be actively transported into cells via ion channels or transport proteins, but due to their high membrane permeability, passive diffusion has been proven to dominate the cellular uptake of even the anionic PFAS.^25^ There is no pH gradient under typical bioassay conditions; thus, even charged organic chemicals reach a steady state in cells within hours.^22^ It is safe to assume that free concentrations in cells (*C*_free,cytosol_) and *C*_free,medium_ are equal at the steady state.

If *C*_lip_ is related to a constant cell membrane concentration of baseline toxicants, namely, IC_10,membrane,baseline_ of 69 mmol/L_lip_,^6^ the *C*_w_ presents the free concentration of baseline toxicants in the aqueous phase of both medium and cells, IC_10,free,baseline_, which can be calculated by transforming eqs 7 to 8.
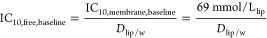
8

### Nominal Concentrations Related to Baseline Toxicity

Combining eqs 6 and 8 yields eq 9, which predicts the nominal baseline toxicity.
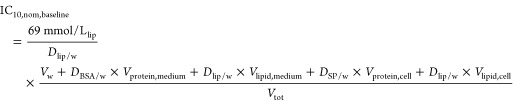
9

There is a linear relationship between proteins (log *D*_BSA/w_ or log *D*_SP/w_) and lipids (log *D*_lip/w_) for nonspecific binding, as shown in eq 10.
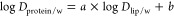
10

Inserting eqs 10 to 9 yields an equation that is dependent only on the log *D*_lip/w_ and system parameters of the bioassay. The model can be fitted by an empirical exponential equation to mathematically simplify the equation to reduce it to three adjustable parameters (eq 11). Previously, Lee et al.^7^ developed an empirical baseline toxicity prediction model for neutral and cationic chemicals. As anionic PFAS bind stronger to proteins than comparable neutral chemicals, the model will need to be updated for anionic PFAS, and there will be a separate baseline toxicity QSAR (quantitative structure–activity relationship) for neutral and anionic PFAS.
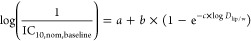
11

### Toxic Ratio and Specificity Ratio

The ratio of cytotoxic concentrations (IC_10_) between predicted baseline toxicity and experimental cytotoxicity is the toxic ratio TR (Figure 1),^8,9^ which can be derived from the free (TR_free_) or the nominal effect concentrations (TR_nom_) according to eq 12.
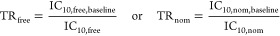
12

In theory, TR_free_ and TR_nom_ are the same, but the empirical values might differ due to different points of departure for their calculations. Chemicals with a TR < 10 are classified as baseline toxicants. TR > 10 suggests that there may be some specific mode of action triggering the toxic effects before baseline toxicity occurs.^8,9^

Any effect concentration (EC) for reporter gene activation or other defined targets can also be related to baseline toxicity by defining a SR, as shown in eq 13.
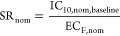
13

F is typically 10%, and EC_10_ is defined as the effective concentration triggering 10% of the maximum effect. For antagonism, the suppression ratio SPR of 20% is often used, and in the present study, EC_SPR20_ is the suppression concentration leading to 20% inhibition of the background signal of rosiglitazone on PPARγ.^23^ SR < 1 suggests that the effects on defined targets may be nonspecific. One ≤ SR < 10 is considered as moderate specificity with uncertainty. SR > 10 is specific.^9^

## Materials and Methods

### Materials

Eleven PFAS (perfluorobutanoic acid (PFBA), perfluorohexanoic acid (PFHxA), perfluoroheptanoic acid (PFHpA), perfluorooctanoic acid (PFOA), perfluorononanoic acid (PFNA), perfluoroundecanoic acid (PFUnA), perfluoro-2-methyl-3-oxahexanoic acid (HFPO–DA), perfluorohexahexanesulfonic acid (PFHxS), perfluorooctanesulfonic acid (PFOS), 6:2 fluorotelomer sulfonic acid (6:2 FTSA), and perfluorooctane sulfonamide (PFOSA)) were investigated in detail. Their structures are shown in Figure S1. Additional 13 PFAS (10 per- and three polyfluorinated chemicals) were evaluated only in the cell-based assays (Table S1). All PFAS were dissolved in methanol (1428, Chemsolute) as a stock solution (Table S2).

### Free Concentration and Cytotoxicity of PFAS in PPARγ-GeneBLAzer Reporter Gene Assays

A detailed description is in Supporting Information text S2, and an experimental workflow is shown in Figure S2. Briefly, on day 1, cells were seeded in a 96-well plate (655946, Greiner) for 24 h of incubation. On day 2, PFAS stock solutions in methanol were pipetted into dosing vials (2214340, Labsolute), and the methanol was blown down gently with nitrogen. The PFAS precipitate at the bottom of each vial was dissolved again with 1000 μL assay medium and serially diluted in a 96-deep-well plate (7696548, Labsolute) with a dilution factor of 2 to obtain 10 concentration points. Then, 120 μL of PFAS dosing medium was transferred from a 96-deep well plate to a cell plate for 24 h of exposure. The concentration exposed to cells is shown in Table S2. On day 3, the cell plate was imaged with the IncuCyte S3 live cell imaging system (Essen BioScience, USA). The cytotoxicity was determined by comparing the confluency of exposed cells and unexposed cells. Then, 200 μL of the supernatant from each well in the cell plate was transferred to a 96-deep-well plate (P-DW-500-C, Labsolute) for SPME to measure the free concentrations of PFAS in the medium. The experimental conditions for Supelco BioSPME 96-Pin Devices (59683-U, Sigma-Aldrich) are listed in Table S3a, and the calculation details for free concentrations of PFAS are as Henneberger et al.^26^

### PPARγ-GeneBLAzer Medium Binding of PFAS

A serial dilution of PFAS in a 96-deep well plate (7696548, Labsolute) was prepared as above. A second 96-well plate (655946, Greiner) was filled with 100 μL of fresh assay medium. 120 μL of PFAS dosing medium was transferred from the 96-deep well plate to the second 96-well plate to make the concentrations of PFAS the same as those exposed to cells (Table S2). Then, 200 μL was transferred to the third 96-deep-well plate (P-DW-500-C, Labsolute) for SPME with a Supel BioSPME 96-Pin device. The distribution ratios of PFAS between medium components and water (*D*_medium/w_) were determined as described by Qin et al.^23^

### Cell Binding of PFAS

The detailed description is in Supporting Information text S3, and the experimental workflow for cell binding experiments is shown in Figure S3. HEK293H cells (modified in the PPARγ-GeneBLAzer reporter gene assay) were homogenized by ultrasonic shattering (Sonoplus 2070, Germany). PFAS stock solutions were diluted with PBS. 100 μL of cell homogenate and 100 μL of PFAS solution were added and vortexed in a 1.5 mL HPLC vial with insert (7648146, 765116, Labsolute). Cell homogenates were derived from approximately 1.25 × 10^6^ cells per experiment, and the concentrations of PFAS are listed in Table S2. We also studied the cell binding of PFAS with the other three cell lines (MCF7, H4lle, and SH-SY5Y), which were used in three cell-based HTS (AREc32, AhR-CALUX, and neurotoxicity). The distribution ratios of PFAS between cells and water (*D*_cell/w_) were analyzed as described by Qin et al.^23^

### Structural Protein Binding of PFAS

The detailed description is in Supporting Information text S4, and the experimental workflow is shown in Figure S4. The structural protein binding assay applied ground powder of chicken breast fillet, which was prepared as described previously.^24^ Chicken protein was suspended with PBS by a high-speed vortex. The pH value of the suspension was adjusted to neutral (pH = 7.4). 500 μL of protein suspension and 500 μL of PFAS solution were added and vortexed in a 1.5 mL HPLC vial (7654554, 7663230, Labsolute). The concentration of protein in each sample was 50 mg/mL, and the concentrations of PFAS are listed in Table S2. The binding affinity of PFAS to cells and structural proteins is considered weak,^24^ and thus, the C18-SPME fiber (57281-U, Sigma-Aldrich) with a larger volume of C18-particles embedded metal alloy was used. The experimental conditions for the C18-SPME fiber are in Table S3b. The distribution ratios of PFAS between structural protein and water (*D*_SP/w_) were analyzed according to Qin et al.^23^

### Protein and Lipid Content of Medium and Cells

Protein concentrations of assay mediums and cell homogenates for four assays were determined by a Pierce BCA Protein Assay Kit (23228, Thermo Scientific). Their lipid concentrations were determined by the sulfo-phospho-vanillin reaction, as described previously.^27^ Units of protein and lipid were converted from mass concentration (mg/L) to volume concentration (mL/L) using a density of protein of 1.36 kg/L and a density of lipid of 1 kg/L.

### High-Throughput Screening of PFAS in 384-Well Plates

An experimental workflow for four cell-based bioassays with different targets (PPARγ-GeneBLAzer, AREc32, AhR-CALUX, and neurotoxicity) is shown in Text S5 and Figure S5. These assays were performed in 384-well plates as described previously.^7,28−30^ Experimental conditions, including assay medium, cell lines, and cell number, are in Table S4, and PFAS concentrations are in Table S5. Briefly, on day 1, cells were seeded in a 384-well plate by a MultiFlo Dispenser (BioTek,Vermont, USA). On day 2, PFAS stock solutions were prepared with methanol. Defined volumes of PFAS stock were transferred to dosing vials (2214340, Labsolute) and blown down with nitrogen. PFAS were dissolved again in the assay medium. Then, serial concentrations of PFAS in a medium were prepared and dosed to cells by Hamilton Star Robot (Bonaduz, Switzerland). Cell plates were imaged by IncuCyte S3 at the start of exposure. On day 3, cell plates were imaged by IncuCyte S3 again after 24 h of PFAS exposure. The cytotoxicity of three reporter gene cell lines was determined by comparing the confluency of exposed cells and unexposed cells. The cell responses targeting PPARγ, AREc32, and AhR were quantified from the signals of reporter proteins with an Infinite M1000 plate reader (Tecan, USA). The neurite length of differentiated SH-SY5Y cells was quantified by phase-contrast imaging using an IncuCyte S3. Then, Nuclear Green LCS1 (ab138904, Abcam) and propidium iodide (81845, Sigma-Aldrich) were used to stain the total cells and death cells, which were quantified with the IncuCyte S3 to determine the cytotoxicity.

## Results and Discussion

### Binding to Medium Components, Cells, Liposomes, and Proteins

*D*_medium/w_ and *D*_cell/w _ of ten anionic PFAS and the partially anionic PFOSA were determined exclusively with PPARγ-medium and the HEK293H cell line. The *D*_medium/w_ values of hydrophilic PFBA, PFHxA, and PFHpA and of the hydrophobic PFUnA were independent of concentration (Figure S6) and were used in the MBM (eq 3) to predict the *C*_free,medium_. In contrast, the *D*_medium/w _ of seven other PFAS (PFOA, PFNA, HFPO–DA, PFHxS, PFOS, 6:2 FTSA, and PFOSA) were found to be concentration-dependent (Figure S6). PFAS-specific regression equations of log *D*_medium/w_ against log *C*_w_ were derived from the Freundlich-type model (Table S6).^23^

As the protein content in the medium was 64 times higher than the lipid content (Table S7), the concentration-dependent protein binding of anionic PFAS dominated the medium binding.^23^ IC_10,nom_ or IC_10,free_ (Table 1) were derived from the concentration–cytotoxicity curve at 10% cytotoxicity with a nominal or free concentration of PFAS in the medium (Figure S7). The log *D*_medium/w_ at IC_10,nom_ was calculated from the regression eqs (Table S6) with measured *C*_free_ at IC_10,free_. log *D*_cell/w_ of HEK293H cells were measured at constant concentrations close to the IC_10,nom_ (Table 1). As the protein and lipid are major sorption phases of the medium component and cells, *D*_BSA/w_, *D*_SP/w_, and *D*_lip/w_ were used as representatives of *D*_medium/w_ and *D*_cell/w_ for the MBM (eq 6). Log *D*_BSA/w_ were calculated with IC_10,free_ from the regression equations listed in Table S6, and log *D*_SP/w_ were measured at concentrations close to IC_10,nom_. log *D*_lip/w_ were collected from literature^25,31^ or predicted from other experimental descriptors (Figure S8, eqs S9–S11).

**Table 1:** Cytotoxicity of 11 PFAS in the PPARγ-GeneBLAzer Assay and Distribution Ratios of PFAS between Different Biomaterials and Water^a^

	DTXSID	IC_10,nom_ [mol/L]	IC_10,free_ [mol/L]	log *D*_medium/w_ [*L*_w_/*L*_prot+lip_]^b^	log *D*_cell/w_ [*L*_w_/*L*_prot+lip_]^c^	log *D*_BSA/w_ [*L*_w_/*L*_prot_]^b^	log *D*_SP/w_ [*L*_w_/*L*_prot_]^c^	log *D*_lip/w_ [*L*_w_/*L*_lip_]	TR_nom_	TR_free_
PFBA	DTXSID4059916	3.19 × 10^–3^	1.78 × 10^–3^	2.60	2.40	1.94	2.11	1.00^d^	2.36	3.88
PFHxA	DTXSID3031862	9.95 × 10^–4^	4.72 × 10^–4^	2.78	3.45	2.70	2.45	2.32^e^	0.49	0.70
PFHpA	DTXSID1037303	3.75 × 10^–4^	6.76 × 10^–5^	3.21	3.69	3.40	2.65	2.91^e^	0.86	1.26
PFOA	DTXSID8031865	1.30 × 10^–4^	1.84 × 10^–5^	3.67	3.94	3.98	3.14	3.52^e^	1.32	1.13
PFNA	DTXSID8031863	9.07 × 10^–5^	7.72 × 10^–6^	4.14	3.74	4.39	3.39	4.25^e^	1.68	0.50
PFUnA	DTXSID8047553	2.16 × 10^–5^	3.83 × 10^–7^	4.74	3.78	4.75	3.97	4.54^e^	5.04	5.20
HFPO–DA	DTXSID70880215	6.54 × 10^–4^	2.20 × 10^–4^	3.18	3.09	2.28	2.77	2.41^e^	0.47	1.22
PFHxS	DTXSID3037709	1.46 × 10^–4^	4.22 × 10^–5^	3.14	3.56	3.52	2.95	4.13^e^	0.17	0.12
PFOS	DTXSID8037706	4.78 × 10^–5^	3.89 × 10^–6^	4.04	4.32	4.52	3.94	4.89^e^	0.30	0.23
6:2 FTSA	DTXSID6067331	3.07 × 10^–4^	4.25 × 10^–5^	3.45	3.90	3.71	3.39	3.87^f^	0.25	0.22
PFOSA	DTXSID3038939	7.85 × 10^–6^	3.28 × 10^–7^	4.18	3.81	4.33	3.52	4.94^f^	2.25	2.43

a Nominal and free inhibitory concentrations of PFAS triggering 10% cytotoxicity (IC_10,nom_ and IC_10,free_). Distribution ratios of PFAS between PPARγ-medium and water (*D*_medium/w_), between HEK293H (PPARγ) cells and water (*D*_cell/w_), between BSA and water (*D*_BSA/w_), and structural protein and water (*D*_SP/w_). Literature and predicted distribution ratios between liposome and water (*D*_lip/w_). Toxic ratios (eq 12) with free (TR_free_) and nominal (TR_nom_) concentrations in the PPARγ-GeneBLAzer assay.

b The concentration-dependent distribution ratios *D*_medium/w_ and *D*_BSA/w_ at IC_10,free_ were derived from the empirical equations given in Table S6.

c log *D*_cell/w_ and log *D*_SP/w_ were measured at PFAS concentrations near IC_10,nom_ (Table S2).

d log *D*_lip/w_ were from Droge et al.^31^

e log *D*_lip/w_ were from Ebert et al.^25^

f log *D*_lip/w_ of 6:2 FTSA and PFOSA was predicted from the linear relationship of experimental log *D*_lip/w_ from literature^25,31^ against the number of fluorinated carbons (Figure S8, eq S10).

### Free and Nominal Concentration of PFAS in the PPARγ-GeneBLAzer Reporter Gene Assay

**Figure 2:**
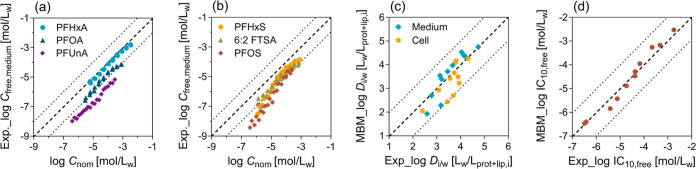
Relationship between nominal (*C*_nom_) and free (*C*_free,medium_) concentrations measured in the PPARγ-GeneBLAzer reporter gene assay for (a) three perfluoroalkyl carboxylic acids and (b) three perfluoroalkyl sulfonic acids. (c) Experimental (Exp) distribution ratios between medium and water (*D*_medium/w_) and cell and water (*D*_cell/w_) of 11 PFAS plotted against predicted *D*_medium/w_ and *D*_cell/w_. *D*_medium/w_ was predicted by eq 4 with experimental distribution ratios between BSA and water (*D*_BSA/w_) and between liposome and water (*D*_lip/w_) (Table 1). *D*_cell/w_ values were predicted by eq 5 with distribution ratios between structural protein and water (*D*_SP/w_) and *D*_lip/w_ (Table 1). (d) Inhibitory concentration IC_10,free_ values were derived from concentration–cytotoxicity curves at 10% cytotoxicity with measured *C*_free,medium_. Experimental (Exp) IC_10,free_ plotted against IC_10,free_ predicted by the MBM (eq 6) with *D*_lip/w_, *D*_BSA/w_, and *D*_SP/w_.

PFAS are considered proteinophilic and lipophilic,^32^ and thus, PFAS are prone to bind to components of medium and cells in bioassay systems, indicating *C*_free,medium_ would be lower than the associated *C*_nom_.^33^ As the numbers of carbons in the three perfluoroalkane carboxylic acids increased, the *C*_free,medium_ deviated more from the 1:1 line to the corresponding *C*_nom_, but the deviation was independent of the concentration (Figure 2a). The measured *C*_free,medium_ values of the more hydrophilic PFHxA were close to *C*_nom_, while the hydrophobic PFUnA had *C*_free,medium_ values up to 90 times lower than *C*_nom_. The relationship between log *C*_free,medium_ and log *C*_nom_ was not linear, which was most pronounced for PFHxS and PFOS (Figure 2b). The difference can be explained because sulfonic acids were found to have specific binding to proteins at low concentration ranges, resulting in a higher deviation of *C*_free,medium_ from *C*_nom_ at low concentrations.

The observed differences between *C*_free,medium_ and *C*_nom_ can be explained by the MBM. The *C*_free,medium_ predicted by the MBM (eq 3) with *D*_medium/w_ (Table S6) and *D*_cell/w_ (Table 1) agreed well with the experimental ones in a concentration-dependent way (Figure S9). *C*_free,medium_ predicted by the MBM (eq 6) with *D*_lip/w_, *D*_BSA/w_, and *D*_SP/w_ were also consistent (Figure S9) because the experimental log *D*_medium/w_ (Table 1) at IC_10_ and were represented well by log *D*_BSA/w_ at IC_10_ and log *D*_lip/w_ (eq 4), as well as the log *D*_cell/w_ with log *D*_sp/w_ and log *D*_lip/w_ (eq 5) in Figure 2c. The volume fractions of proteins and lipids in the medium and cells are shown in Tables S7 and S8. Consequently, the experimental and predicted IC_10,free_ values by the MBM (eq 6) also agreed well (Figure 2d).

### Toxic Ratios: Baseline Toxicity and Cytotoxicity of PFAS

**Figure 3:**
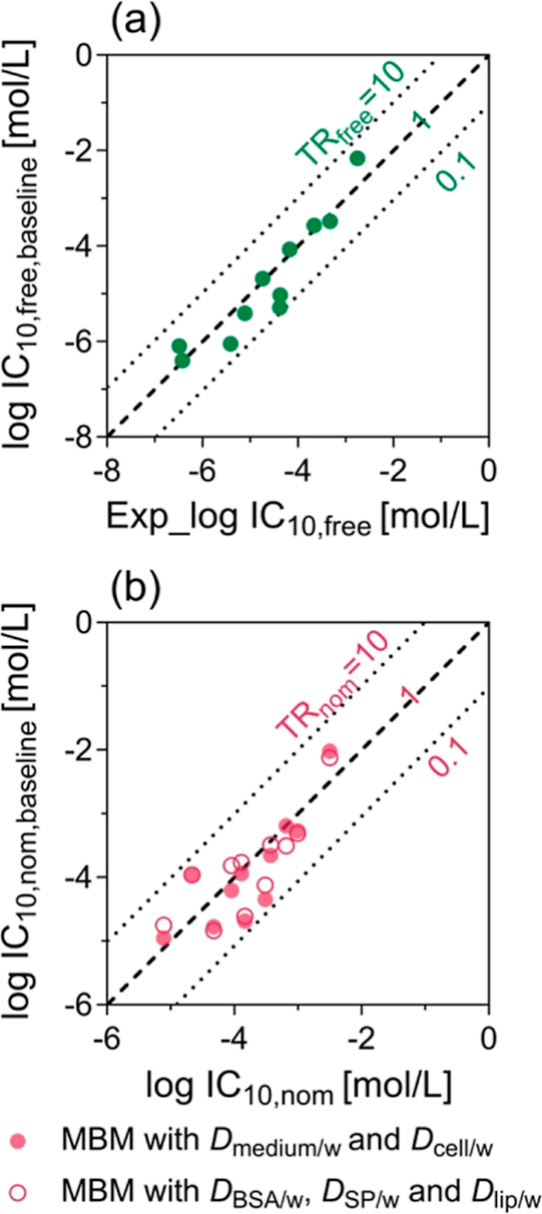
TR (eq 12) of PFAS in the PPARγ-GeneBLAzer assay. (a) Measured free concentration at 10% cytotoxicity IC_10,free_ (Table 1) plotted against the IC_10,free,baseline_ predicted from the critical membrane burden of 69 mmol/L_lip_ (eq 8). The diagonal line depicts the TR_free_ of 1, and the dotted lines represent the range for the baseline toxicity of 10 > TR_free_ > 0.1. (b) Nominal concentration at 10% cytotoxicity IC_10,nom_ plotted against the IC_10,nom,baseline_ predicted from IC_10,free,baseline_ by eq 3 (filled circle) or eq 6 (empty circle). The diagonal line depicts the TR_nom_ of 1, and the dotted lines represent the range for the baseline toxicity of 10 > TR_nom_ > 0.1.

The toxic ratio TR (eq 12) allows an estimation if PFAS act as baseline toxicants or exert cytotoxicity due to a specific effect. All experimental IC_10,free_ (Table 1) values were close to the IC_10,free,baseline_ values (Table S9) predicted from the critical membrane concentration of 69 mmol/L_lip_ (eq 8). TR_free_ of the 11 PFAS were within the range of baseline toxicants of 0.1–10, suggesting that the cytotoxicity of these PFAS is caused by baseline toxicity (diagonal lines in Figure 3a).

TR_nom_ is more practical than TR_free_ because nominal concentrations are widely reported in the literature. IC_10,nom,baseline_ (Table S9) were predicted from IC_10,free,baseline_ by eqs 3 and 6 with the experimental parameters in Table 1. Consistently, the TR_nom_ of the PFAS was still within the range of 0.1–10 (Figure 3b), confirming that PFAS behave like baseline toxicants in the cytotoxicity endpoint of the PPARγ-GeneBLAzer assay.

### Development of a Baseline Toxicity Prediction Model for Nominal Cytotoxicity

Log *D*_BSA/w_ and log *D*_SP/w_ are both linearly correlated (eq 10) against log *D*_lip/w_ (Table 1) for the nine anionic PFAS (Figure S10a,b). 6:2 PFSA and PFOSA were excluded from the regressions in eqs 14 and 15 because their log *D*_lip/w_ were only predicted, but they are plotted in Figure S10a,b for comparison.
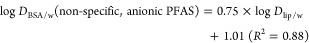
14
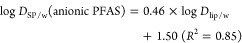
15

Previously, a relationship of log *D*_BSA/w_ against log *D*_lip/w_ for neutral chemicals was derived by Endo and Goss^34^ (eq 16, rescaled from *K*_ow_). The higher affinity of anionic PFAS for proteins is evident by the larger slope and intercept in eq 14 than in eq 16. The binding of anionic PFAS to structural proteins (eq 15) was also stronger than that of neutral chemicals (eq 17)^35^ for log *D*_lip/w_ < 5.
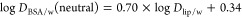
16
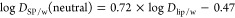
17

Insertion of eqs 14 and 15 in eq 9 leads to eq 18 with *sD*_lip/w_ as the sole input parameter to derive baseline toxicity predictions for anionic PFAS. The volume of cells is much lower than the volume of the medium, and the volume fraction of protein and lipid in the medium is <1% (Tables S7 and S8). Therefore, *V*_tot_ ≈ *V*_medium_ ≈ *V*_w_ and the volume fraction (Vf) of protein and lipid in the medium and cells were used to present the distributions of protein and lipid in the baseline toxicity prediction model.
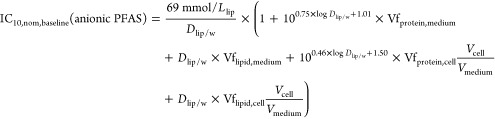
18

For comparison, a baseline toxicity prediction model for neutral chemicals was developed by the insertion of eqs 16 and 17 into eq 9.
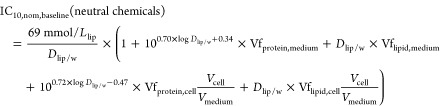
19

**Figure 4:**
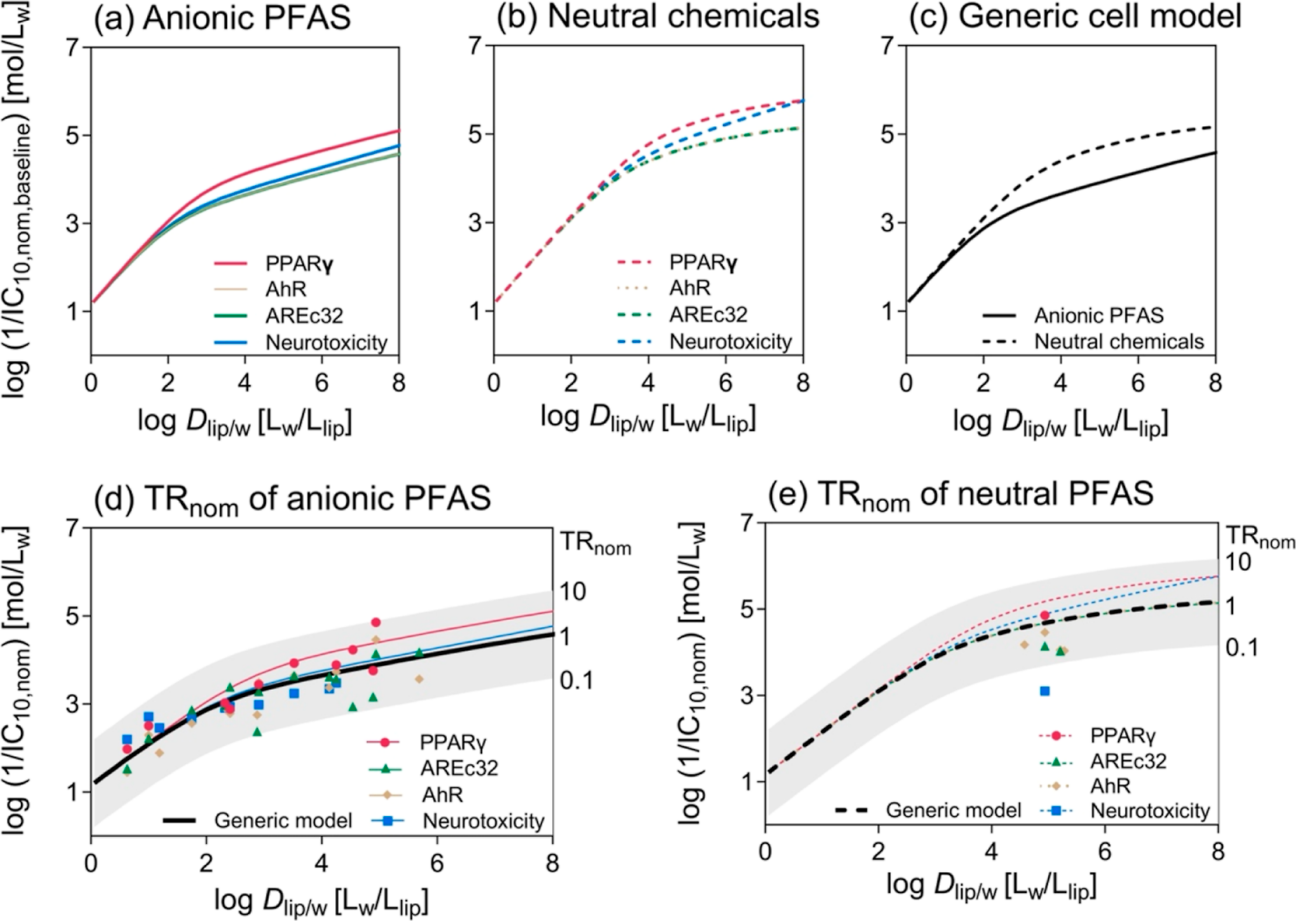
Baseline toxicity prediction models for (a) anionic PFAS (eq 18) and (b) neutral chemicals (eq 19) in four cell-based bioassays: PPARγ-GeneBLAzer (magenta), AREc32 (green), AhR CALUX (gold), and neurotoxicity (blue). (c) Baseline toxicity prediction models with the generic cell model (eqs 18, 19). (d) Nominal inhibitory concentration at 10% cytotoxicity IC_10,nom_ of 16 anionic PFAS in four cell-based bioassays: PPARγ (magenta circle), AREc32 (green triangle), AhR (gold diamond), and neurotoxicity (blue square) and color-matched bioassay-specific baseline toxicity prediction models as well as the generic model (black line). The gray area depicts the range for baseline toxicity of 10 > TR_nom_ > 0.1. (e) IC_10,nom_ of partially charged and neutral PFAS in four cell-based bioassays compared with the baseline toxicity prediction models as well as the generic model (black broken line). All fit parameters of the empirical baseline toxicity prediction model (eq 11) are listed in Table 2.

Analogously to Lee et al.,^7^ empirical baseline toxicity prediction models for anionic PFAS were derived for all four cell lines by inserting bioassay-specific volumes of protein and lipids in cells and medium measured in the present study (Tables S7 and S8) into eq 18. The models differ because the protein and lipid contents in the assay systems are different. The resulting predictions for anionic PFAS are depicted in Figure 4a. It can be expected that any anionic chemicals behave similarly to anionic PFAS, but as the model was calibrated with anionic PFAS data only, we consider it a PFAS-specific model. The model holds for neutral chemicals (Figure 4b) in general and includes neutral PFAS. These bioassay-specific empirical baseline toxicity models for neutral chemicals and anionic PFAS for the four cell lines were derived by exponential fit (eq 11), and the adjustable parameters *a*, *b*, and *c* are listed in Table 2.

**Table 2:** Parameters of the Empirical Baseline Toxicity Prediction Model (eq 11) of Anionic and Neutral PFAS in Four Cell-Based Bioassays and the Generic Cell Assay

		Anionic PFAS			Neutral PFAS		
assay	Medium	*a*	*b*	*c*	*a*	*b*	*c*
PPARγ (HEK293H)	OptiMEM +2 % FBS	1.25	4.76	0.251	1.24	5.47	0.235
AREc32 (MCF7)	DMEM Glutamax + 10% FBS	1.22	3.78	0.263	1.26	4.43	0.278
AhR (H4IIe)	DMEM Glutamax + 10% FBS	1.22	3.78	0.263	1.26	4.45	0.277
neurotoxicity (SH-SY5Y)	neurobasal medium	1.22	4.07	0.247	1.23	5.61	0.209
generic cell (6% proteins, 0.1% lipids)	generic medium (0.3% proteins, 0.001% lipids)	1.22	3.79	0.262	1.26	4.47	0.275

If the baseline toxicity models were used to evaluate literature bioassay data with undisclosed conditions, we assumed the volume fractions of protein and lipid in medium are Vf_protein,medium_ of 3 mL_protein_/L_medium_ and Vf_lip,medium_ of 0.07 mL_lipid_/L_medium_ (Table S7), and Vf_protein,cell_ is 30 mL_protein_/L_cell_ and Vf_lip,cell_ is 5 mL_lipid_/L_cell_ (Table S8). The volume of medium *V*_medium_ and volume of cells *V*_cell_ are dependent on microtiter plates in 96-, 384-, or 1536-well formats, e.g., *V*_medium_ of 40 μL and *V*_cell_ of approximately 30 nL for the 384-well plate format. The fit parameters of this generic bioassay model are also listed in Table 2.

These two models are compared in Figure 4c, where the two lines started to separate at log *D*_lip/w_ > 2. This is due to anionic chemicals having a higher affinity for proteins despite baseline toxicity occurring at concentrations where the protein binding is dominated by nonspecific binding. At log *D*_lip/w_ < 2 (e.g., PFBA), IC_10,nom_ is close to IC_10,free_ (Table 1 and Figure S9a). Therefore, eqs 18 and 19 are simplified to eq 20, which is similar to eq 8 and is valid for neutral, anionic, and cationic organic chemicals.
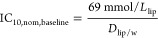
20

Sensitivity analysis was performed to study how various parameters contribute to the baseline toxicity prediction model. The protein and lipid contents of these four cell lines were similar (Table S8), and thus, the *D*_cell/w_ of single PFAS (Table S10) among the four cell lines did not differ much. Besides, the ratio of *V*_cell_/*V*_medium_ is usually <0.001. Therefore, the differences between baseline toxicity prediction models (eqs 18 and 19) with cells and without cells are negligible (Figure S10c).

By contrast, medium components make a difference. Models for AREc32 and AhR CALUX assays were overlaying because the same medium was used in the assays, while less protein and lipid were in the media for PPARγ-GeneBLAzer and neurotoxicity assays (Table S7). The difference is more pronounced as the chemical hydrophobicity increases (Figure 4a,b). The lipid-bound fraction is smaller compared to protein, but it still influences the predicted IC_10,nom,baseline_. For example, if Vf_lip,medium_ changed from 0.07 mL/L to 0, the model for anionic PFAS did not change much because the concentration of anionic PFAS bound to protein is much higher than that bound to lipid, but the model for neutral chemicals deviates for chemicals with log *D*_lip/w_ > 4 (Figure S10d). We recommend to measure the volume fractions of protein and lipid in the medium used for bioassays on a routine basis as they are the main determinants of the model.

### Is the Cytotoxicity of PFAS Merely Baseline Toxicity?

24 PFAS (Table S1) were measured in this study. These PFAS have diverse functional groups, including sulfonic acid, carboxylic acid, oxide dimer acid, fluorotelomer alcohol, and sulfonamide, as well as some fluorinated pesticides that contain individual C_*n*_F_2*n*_-groups. Their log *D*_lip/w_ ranges over 6 orders of magnitude (Table S11). Log *D*_lip/w_ values of these PFAS were used in the baseline toxicity prediction model to derive the IC_10,nom,baseline_ (eq 11).

The IC_10,nom_ of 24 PFAS were measured in four bioassays in 384-well plates (Table S12). The IC_10,nom,baseline_ of 15 anionic PFAS were predicted with eq 18, while IC_10,nom,baseline_ of the other four partially charged and five neutral PFAS were predicted with eq 19. As shown in Figure 4d,e, the measured IC_10,nom_ were within a factor of 10 to the IC_10,nom,baseline_ predicted by the four bioassay-specific models and all well within the gray band of 10 > TR_nom_ > 0.1 of the generic model, indicating that these PFAS did not show specific effects but baseline toxicity was the cause of their cytotoxicity. The generic cell model is adequate to provide IC_10,nom,baseline_ values as a reference for general experimental conditions.

### Application of the Baseline Toxicity Prediction Model to Evaluate the Specificity of Effects

**Figure 5:**
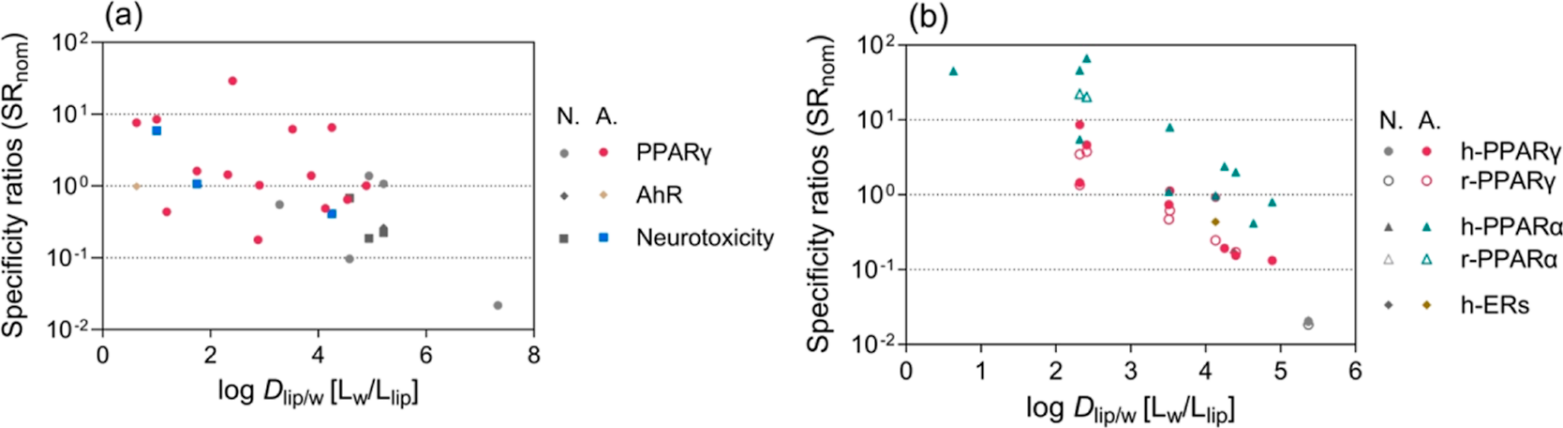
Specificity ratios SR_nom_ (eq 13) of PFAS in cell-based bioassays. (a) SR of 16 anionic (A.) and eight neutral (N.) PFAS in cell-based bioassays of PPARγ, AhR, and neurotoxicity were measured in this study. (b) SR_nom_ of 11 anionic, one partially charged, and four neutral PFAS in cell-based bioassays of human (h) PPARγ, rat (r) PPARγ, hPPARα, rPPARα, and hERs from Evans et al.^36^

The measured EC_10,nom_ values of the agonistic effects on PPARγ, AREc32, AhR, and neurotoxicity, as well as the EC_SPR20,nom_ values of the antagonistic effects on PPARγ, are listed in Table S12, which were derived from the concentration–response curves of the 24 PFAS investigated experimentally in this study (Figure S11). No activation of the oxidative stress response was detected for all 24 PFAS in AREc32 before cytotoxicity started to kick in. A few PFAS showed weak activation of AhR and neurotoxicity. In contrast, PPARγ was a specific target for 19 of the 24 PFAS (Table S12), but the specificity was low. If the EC_10,nom_ or EC_SPR20,nom_ were compared with the IC_10,nom,baseline_ from anionic or neutral baseline toxicity prediction models for PPARγ, AhR, and neurotoxicity (Table 2), as in Figure 5a, SR_nom_ (eq 13) of most PFAS were within 0.1–10, indicating the effects of PFAS in the tested assays are nonspecific and caused by baseline toxicity. Only HFPO–DA had a SR_nom_ > 10 for the agonistic mode of the PPARγ assay, indicating that HFPO–DA may be a specific PPARγ antagonist.

### Evaluation of Literature Data for Specificity of Effects

Evans et al.^36^ studied the agonistic effects of 16 PFAS (Table S13) on five defined targets from humans or rats, including hPPARγ, rPPARγ, hPPARα, rPPARα, and human estrogen receptors (hER). The IC_10,nom,baseline_ of 16 PFAS was calculated by the generic model (Table 2) from their *D*_lip/w_ (Table S11). Their EC_10,nom_ were recalculated from a linear portion of concentration–response curves because most of the effects were lower than 50% and the linear model is more suitable to derive 10% effects than a four-parameter logistic model.^37^ hPPARα was more likely to be activated than rPPARα (Figure 5b). HFPO–DA, HFPO–DA-AS, and PFMOAA showed SR_nom_ > 10, while the other 13 PFAS either had no effects or had 0.1 < SR_nom_ < 10 and were therefore classified as baseline toxicants. The literature data set showed a clear tendency that SR_nom_ decreased with increasing hydrophobicity (Figure 5b). This phenomenon can be explained by hydrophobic PFAS with strong affinity to membranes, which they may mostly accumulate in the cell membrane before reaching any intracellular-specific target sites. The FTOHs should be excluded from the analysis as they might have escaped from the well-plates or cross-contaminated neighboring wells because they are semivolatile.^6,38,39^

430 PFAS have been selected for a series of bioassay tests in the Tox21/ToxCast program with hundreds of targets (https://comptox.epa.gov/dashboard/chemical-lists/EPAPFASINV). The baseline toxicity prediction model of the present study can be used to evaluate the specificity of different targets once raw data from these bioassays becomes publicly available. Those PFAS with high values of TR and SR on defined targets may provide some hints on initial molecular events and key events, which will facilitate the in vitro to in vivo extrapolation to adverse outcomes and thus promote the development of AOP related to PFAS. However, current results of cell-based HTS from EPA suggest that a majority of 160 PFAS were inactive or equivocal.^18−21^

Although some PFAS showed specific effects on defined targets, their intrinsic specificities appear to be rather low. This does not mean that specific effects are absent. There are many reports about the specific effects of PFAS, but if they are not selective but occur at concentrations similar to baseline toxicity, these effects are easily predictable by the baseline toxicity prediction models derived here. Even if individual PFAS are merely baseline toxicants, their potency can still be of concern because baseline toxicants also cover several orders of magnitude in effect potency.

The biological effects of PFAS were usually detected at the micromolar level in toxicological studies. The blood concentrations of PFAS in some workers and residents living near fluorochemical plants have already reached such levels,^40^ even though that in the general population was at a nanomolar level.^41^ PFAS are highly persistent and have been termed “forever chemicals”. If their production is not stopped, they will continue to build up over time and may eventually reach levels where they cause effects, especially under chronic exposure, where PFAS are known to adversely affect the immune system^42^ and cause liver cancer,^43^ as well as other health impacts.^44,45^ Baseline toxicants act concentration-additive in mixtures, which means that not only the thousands of PFAS but also all other organic chemicals act together in mixtures. Mixture effects may lead to visible effects even if the individual PFAS’ concentrations are below their individual effect threshold.

## Supporting Information Available

Addition of information on chemicals, deviation of equations, experimental procedures and details, volume fractions of proteins and lipids in biomaterials, binding isotherms and binding constants of PFAS between biomaterials and water, a comparison of free and nominal concentrations of PFAS in the bioassay, all results from cell-based HTS assays, and concentration–response curves of 24 PFAS in the PPARγ-GeneBLAzer reporter gene assay (PDF)
