# Supplementary material for: Baseline Toxicity Model to Identify the Specific and Nonspecific Effects of Per- and Polyfluoroalkyl Substances in Cell-Based Bioassays
Source: Environ Sci Technol. 2024 Feb 23;58(13):5727–38. doi: 10.1021/acs.est.3c09950 (PMC10993398; doi:10.1021/acs.est.3c09950)
Supplement: Supplementary file 1 — es3c09950_si_001.pdf [file es3c09950_si_001.pdf]

Supporting information

Baseline Toxicity Model to Identify the Specific and Non-specific Effects of Per- and Polyfluoroalkyl Substances in Cell-based Bioassays

Weiping Qin,[1,2] Luise Henneberger,[1] Juliane Glüge,[1,3] Maria König[1] and Beate I. Escher[1,2]*

[1] Department of Cell Toxicology, UFZ–Helmholtz Centre for Environmental Research, 04318 Leipzig, Germany

[2] Environmental Toxicology, Department of Geosciences, Eberhard Karls University Tübingen, Schnarrenbergstr. 94-96, DE-72076 Tübingen, Germany

[3] Institute of Biogeochemistry and Pollutant Dynamics, ETH Zürich, 8092 Zürich, Switzerland.

*Corresponding author Beate I. Escher − UFZ−Helmholtz Centre for Environmental Research, 04318 Leipzig, Germany; orcid.org/0000-0002-5304-706X; Email: beate.escher@ufz.de

Number of pages: 30

Table of content

- **TEXT: 5**

Text S1. Derivation of Eq. 3 in the main manuscript.

Text S2. Experimental procedure of measuring free concentration and cytotoxicity of PFAS in PPARγ-GeneBLAzer reporter gene assays.

Text S3. Experimental procedure of measuring cell binding assay of PFAS.

Text S4. Experimental procedure of structural protein binding assay of PFAS.

Text S5. Experimental procedure of high throughput screening of PFAS in 384-well plates.

- **TABLES: 13**

Table S1. Purchase information of 24 PFAS used in cell-based bioassays.

Table S2. Total concentrations C_{tot} of 11 PFAS measured by LCMS.

Table S3. Experimental conditions of (a) BioSPME 96-Pin Device for the medium binding assay and (b) the C18-SPME fiber for cell and structural protein binding assay.

Table S4. Information on (a) cells and (b) medium used for the four cell-based HTS bioassays.

Table S5. Maximum concentrations of 24 PFAS in four cell-based HTS bioassays.

Table S6. Distribution ratios between medium and water ($D_{medium/w}$) and distribution ratios between BSA and water ($D_{BSA/w}$) of 11 PFAS.
Table S7. Volume fractions of protein and lipid in medium used in the four cell-based HTS bioassays.
Table S8. Volume fractions of protein and lipid in cells used in the four cell-based HTS bioassays.
Table S9. Free and nominal concentrations of 11 PFAS related to baseline toxicity.
Table S10. Distribution ratios between cell and water ($D_{cell/w}$) of 11 PFAS with four cell lines.
Table S11. Chemical information of anionic and neutral PFAS.
Table S12. Cell responses of 24 PFAS in four cell-based HTS bioassays.
Table S13. Maximum concentration and cell responses of 16 PFAS in five cell-based bioassays.

- **FIGURES: 11**

Figure S1. Structures of 11 PFAS.
Figure S2. Experimental workflow of BioSPME 96-Pin Device used in PPARγ-GeneBLAzer reporter gene assay to measure distribution ratio of PFAS between medium and water ($D_{medium/w}$) and free concentration ($C_{free,medium}$) of PFAS, as well as the inhibitory concentration at 10% cytotoxicity (IC_{10}).
Figure S3. Experimental workflow of C18-SPME used in cell binding assays to measure distribution ratio of PFAS between cell and water ($D_{cell/w}$).
Figure S4. Experimental workflow of C18-SPME used in structural protein binding assays to measure distribution ratio of PFAS between structural protein and water ($D_{SP/w}$).
Figure S5. Experimental workflow of high throughput screening of PFAS in 384-well plates.
Figure S6. Medium binding isotherms of 11 PFAS.
Figure S7. Cytotoxicity of 11 PFAS in the PPARγ-GeneBLAzer reporter gene assay.
Figure S8. Information of lipid and protein binding and PFAS structures.
Figure S9. Relationship between nominal (C_{nom}) and measured free ($C_{free,medium}$) concentrations of PFAS in the PPARγ-GeneBLAzer assay.
Figure S10. Relationships of protein and lipid binding and distribution of medium and cells in the baseline toxicity prediction models.
Figure S11. Agonistic mode and antagonistic mode of PPARγ-GeneBLAzer reporter gene assays of 24 PFAS.

Text S1. Derivation of Eq. 3 in the main manuscript.

The nominal concentration in the medium is the total molar amount of chemicals (n_{tot}) divided by the total volume (V_{tot}), which is composed of medium (V_{medium}) and cells (V_{cell}) (Eq. S1). The free concentration $C_{free,medium}$ is the concentration in the aqueous phase of the medium (Eq. S2).

$$C_{nom}=\frac{n_{tot}}{V_{medium}+V_{cell}}=\frac{n_{tot}}{V_{tot}} \tag{S1}$$

$$C_{free,medium}=\frac{n_{free,medium}}{V_{w,medium}} \tag{S2}$$

The $C_{free,medium}$ relates to C_{nom} by Eq.S3. $f_{free,medium}$ is the fraction of freely dissolved chemical in the medium.

$$C_{free,medium}=C_{nom}\times\frac{n_{free,medium}}{\mathrm{n_{tot}}}\times\frac{V_{tot}}{V_{w,medium}}=C_{nom}\times f_{free,medium}\times\frac{V_{tot}}{V_{w,medium}} \tag{S3}$$

$f_{free,medium}$ is defined by a mass balance equation (Eq. S4), where chemicals are bound to components of medium ($n_{bound,medium}$) and cell ($n_{bound,cell}$) in the bioassay system.

$$f_{free,medium}=\frac{n_{free,medium}}{n_{tot}}=\frac{n_{free,medium}}{n_{free,medium}+n_{bound,medium}+n_{free,cell}+n_{bound,cell}}=\frac{1}{1+\frac{n_{bound,medium}}{n_{free,medium}}+\frac{n_{free,cell}}{n_{free,medium}}+\frac{n_{bound,cell}}{n_{free,medium}}} \tag{S4}$$

Protein and lipid in the medium and cells are the major sorptive phases. The distribution ratio between medium and water ($D_{medium/w}$) is defined by concentration of chemical bound to the protein and lipid in the medium ($C_{bound,medium}$) divided by the freely dissolved concentration in the medium $C_{free,medium}$ as Eq. S5.

$$D_{medium/w}=\frac{C_{bound,\ medium}}{C_{free,medium}}=\frac{n_{bound,medium}}{n_{free,medium}}\times\frac{V_{w,medium}}{V_{protein+lipid,medium}} \tag{S5}$$

Analogously, the distribution ratio between cell and water ($D_{cell/w}$) can be calculated by Eq. S6. At steady state, the free concentration in the cytosol $C_{free,cell}$ can be assumed to be equal to the $C_{free,medium}$.

$$D_{cell/w}=\frac{C_{bound,\ cell}}{C_{free,cell}}=\frac{C_{bound,\ cell}}{C_{free,medium}}=\frac{n_{bound,cell}}{n_{free,medium}}\times\frac{V_{w,medium}}{V_{protein+lipid,cell}} \tag{S6}$$

Inserting Eqs. S5 and S6 in Eq. S4 yields Eq. S7.

$$f_{free,medium}=\frac{V_{w,medium}}{(V_{w,medium}+V_{w,cell})+D_{medium/w}\times V_{protein+lipid,medium}+D_{cell/w}\times V_{protein+lipid,cell}} \tag{S7}$$

Inserting Eq. S7 in Eq. S3 yields Eq. S8. We can simplify $V_w = V_{w,medium} + V_{w,cell}$.

$$C_{free,medium}=C_{nom}\times V_{tot}\times(V_w+D_{medium/w}\times V_{protein+lipid,medium}+D_{cell/w}\times \mathrm{V}_{protein+lipid,cell})^{-1} \tag{S8}$$

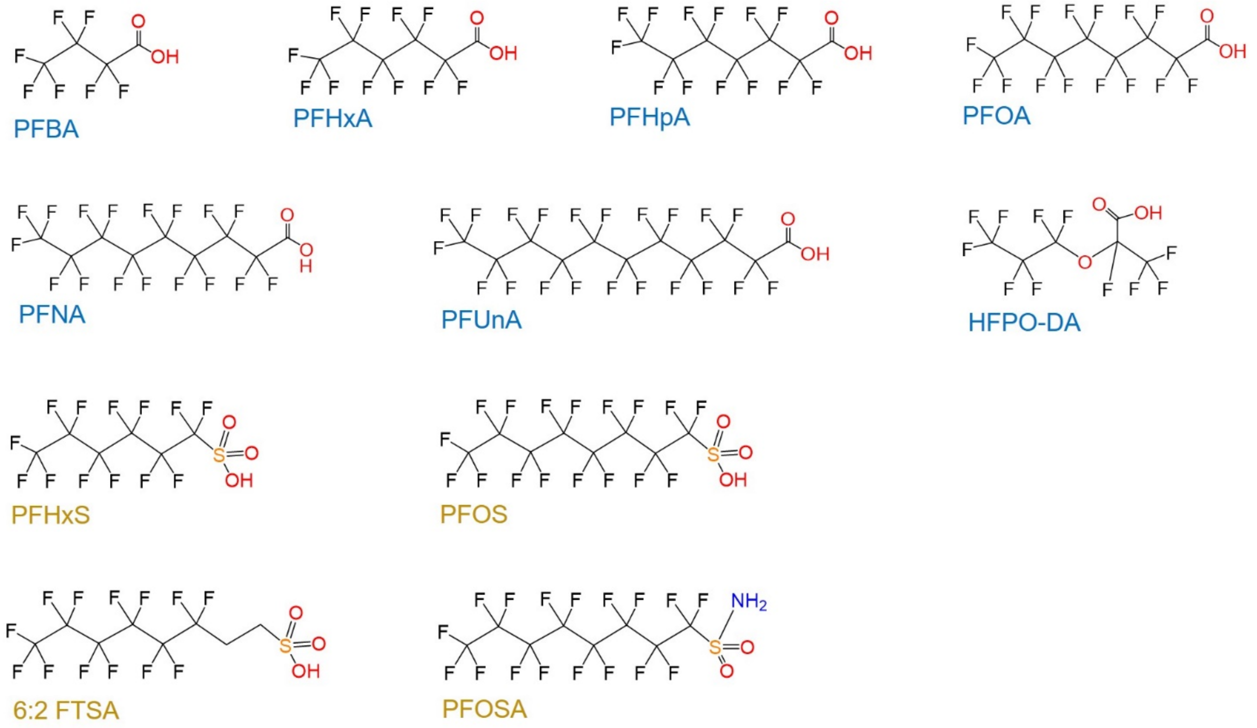

Figure S1. Structures of 11 PFAS.

Table S1. Purchase information of 24 PFAS used in cell-based bioassays.

	DTXSID	Name	Abbreviation	Supplier
**1**	DTXSID8059970	Perfluoropropanoic acid	PFPrA	J&K Scientific
**2**	DTXSID4059916	Perfluorobutanoic acid	PFBA	J&K Scientific
**3**	DTXSID6062599	Perfluoropentanoic acid	PFPeA	J&K Scientific
**4**	DTXSID3031862	Perfluorohexanoic acid	PFHxA	Sigma Aldrich
**5**	DTXSID1037303	Perfluoroheptanoic acid	PFHpA	Sigma Aldrich
**6**	DTXSID8031865	Perfluorooctanoic acid	PFOA	abcr GmbH
**7**	DTXSID8031863	Perfluorononanoic acid	PFNA	chemPUR
**8**	DTXSID8047553	Perfluoroundecanoic acid	PFUnA	chemPUR
**9**	DTXSID8031861	Perfluorododecanoic acid	PFDoDA	Alfa Aeser
**10**	DTXSID00369821	Perfluorooctanedioic acid	PFOdA	chemPUR
**11**	DTXSID70880215	Perfluoro-2-methyl-3-oxahexanoic acid	HFPO-DA	abcr GmbH
**12**	DTXSID2044397	Trifluoromethanesulfonic acid	TfOH	J&K Scientific
**13**	DTXSID3037709	Potassium perfluorohexane sulfonate	PFHxS	Sigma Aldrich
**14**	DTXSID8037706	Potassium perfluorooctane sulfonate	PFOS	Sigma Aldrich
**15**	DTXSID6067331	6:2 Fluorotelomer sulfonic acid	6:2 FTSA	abcr GmbH
**16**	DTXSID3038939	Perfluorooctane sulfonamide	PFOSA	HPC Standards GmbH
**17**	DTXSID1032646	N-Ethylperfluorooctane sulfonamide	nEt-PFOSA	HPC Standards GmbH
**18**	DTXSID6027426	N-Ethyl-N-(2-hydroxyethyl)perfluorooctane sulfonamide	nEt-PFOSE	abcr GmbH
**19**	DTXSID5044572	2-(Perfluorohexyl)ethanol	6:2 FTOH	Sigma Aldrich
**20**	DTXSID7029904	2-(Perfluorooctyl)ethanol	8:2 FTOH	Sigma Aldrich
**21**	DTXSID2029905	2-(Perfluorodecyl)ethanol	10:2 FTOH	J&K Scientific
**22**	DTXSID3032620	Hexaflumuron	Hexaflumuron	HPC Standards GmbH
**23**	DTXSID5034357	Lufenuron	Lufenuron	HPC Standards GmbH
**24**	DTXSID4047672	Flubendiamide	Flubendiamide	HPC Standards GmbH

Table S2. Total concentrations C_{tot} of 11 PFAS measured by LCMS. C_{tot} were used in medium for the PPARγ-GeneBLAzer reporter gene assay, in cell homogenates for the cell binding assay and in chicken protein suspension for structural protein binding as shown in Figure S2, S3 and S4.

Abbreviation	Nominal stock concentration in methanol [mol/L]	Bioassay: C_{tot} in medium exposed to cells [mol/L]	Cell binding assay: C_{tot} in cell homogenate [mol/L]	Structural protein binding assay: C_{tot} in chicken protein suspension [mol/L]
**PFBA**	2.30×10^{-1}	8.65×10^{-3}	2.81×10^{-3}	2.87×10^{-3}
**PFHxA**	9.91×10^{-2}	3.50×10^{-3}	9.30×10^{-4}	1.18×10^{-3}
**PFHpA**	4.75×10^{-2}	1.53×10^{-3}	2.78×10^{-4}	2.14×10^{-4}
**PFOA**	4.75×10^{-2}	1.52×10^{-3}	2.76×10^{-4}	1.71×10^{-4}
**PFNA**	2.50×10^{-2}	1.16×10^{-3}	6.61×10^{-5}	3.41×10^{-5}
**PFUnA**	5.33×10^{-3}	2.89×10^{-4}	4.75×10^{-5}	7.64×10^{-5}
**HFPO-DA**	8.79×10^{-2}	2.01×10^{-3}	2.01×10^{-4}	2.52×10^{-4}
**PFHxS**	2.19×10^{-2}	6.26×10^{-4}	2.39×10^{-4}	2.15×10^{-4}
**PFOS**	8.33×10^{-3}	2.53×10^{-4}	2.09×10^{-5}	4.46×10^{-5}
**6:2 FTSA**	2.44×10^{-2}	7.17×10^{-4}	2.20×10^{-4}	2.43×10^{-4}
**PFOSA**	3.40×10^{-3}	1.11×10^{-4}	2.46×10^{-5}	2.59×10^{-5}

Text S2. Experimental procedure of measuring free concentration and cytotoxicity of PFAS in PPARγ-GeneBLAzer reporter gene assays.

There are four steps in this experiment. Step1: Cell seeding. Cells were seeded in a 96-well plate (655946, Greiner) with volume of 100 µL/well and a cell number of 25000 cells/well.

Step 2: PFAS dosing. Stock solutions of 11 PFAS were prepared with methanol (Table S2) and 50µL stock solution were pipetted to dosing vials (2214340, Labsolute) and blown down gently with nitrogen. The PFAS precipitate at the bottom of each vial was dissolved again with 1000 µL assay medium. The PFAS in the dosing vial were serially diluted in a 96-deep-well plate (7696548, Labsolute) with a dilution factor of 2 to obtain 10 concentration points. Then, 120 µL PFAS dosing medium were transferred from 96-deep well plate to cell plate by a multichannel pipette. The total volume was 220 µL/well. The residual PFAS in the dosing vials were later diluted with PBS to an appropriate concentration before being measured by LCMS to determine the total molar amount (n_{tot}), which was used to calculate the total concentration (C_{tot}) exposed to cells as listed in Table S2.

Step 3: Free concentration of PFAS. The Supelco BioSPME 96-Pin Device was used to measure the free concentration of PFAS. 200 µL supernatant of each well in the cell plate were transferred to a 96-deep-well plate (P-DW-500-C, Labsolute) for SPME. The BioSPME was conditioned in isopropanol (34967, Honeywell) for 20 min and then in MilliQ water for 10 s before chemical extraction. The extraction and desorption processes were performed on a high-speed shaker (Bioshaker, Qinstruments, Germany) with a shaking speed of 1000 rpm for 60 min. Desorption solvent were a mix of 50% MeOH and 50% MilliQ water for 6 hydrophilic PFAS or 100% MeOH for 5 hydrophobic PFAS. The temperature of the Bioshaker was set to 37°C for extraction but for the desorption was reduced to 20°C to avoid the evaporation of solvent. PFAS in the desorption solvent were measured by a 1260 Infinity liquid chromatograph coupled with 6420 Triple Quad mass spectrometer (LCMS, Agilent, USA). The experimental conditions are listed in Table S3a. and calculation details for free concentrations of PFAS in the medium is as Henneberger et al.[1]

Step 4: Cytotoxicity. Cell plate was imaged with IncuCyte S3 after dosing with PFAS. The cytotoxicity was determined by comparing the confluency of exposed cells and un-exposed cells after 24 h PFAS exposure.

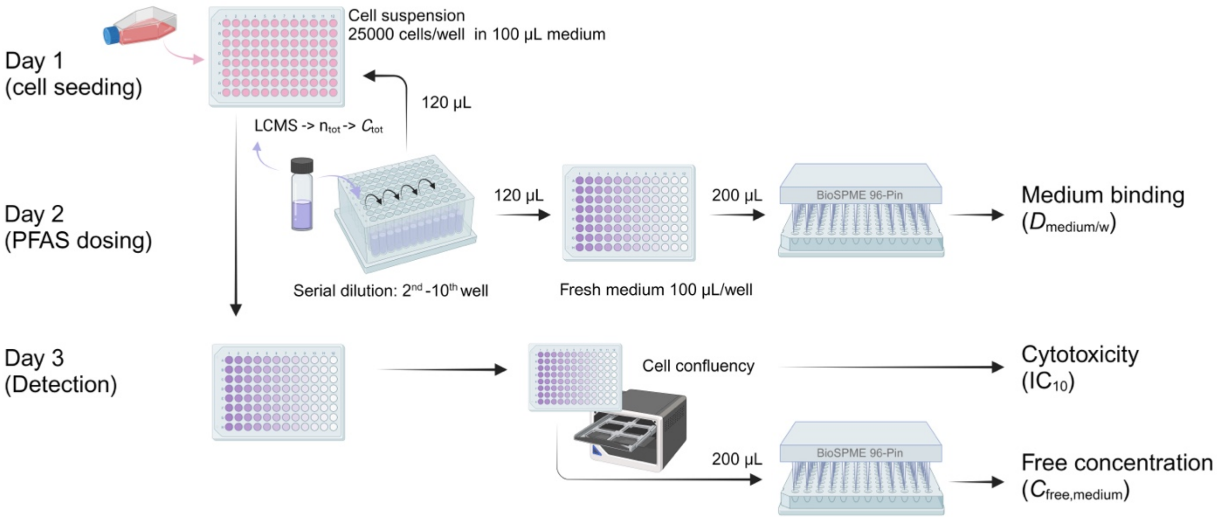

Figure S2. Experimental workflow of BioSPME 96-Pin Device used in PPARγ-GeneBLAzer reporter gene assay to measure distribution ratio of PFAS between medium and water ($D_{\text{medium/w}}$) and free concentration ($C_{\text{free,medium}}$) of PFAS, as well as the inhibitory concentration at 10% cytotoxicity (IC_{10}).

Table S3. Experimental conditions of (a) BioSPME 96-Pin Device for the medium binding assay and (b) the C18-SPME fiber for cell and structural protein binding assay.

(a)	C18-coating volume	Assay plate	Condition for extraction	Condition for desorption	Desorption solvent
PFBA	80 nL	96-deep well plate [600µL]	60 min, 1000 rpm, 37℃	60 min, 1000 rpm, 20℃	50% Methanol+50% MilliQ water
PFHxA					50% Methanol+50% MilliQ water
PFHpA					50% Methanol+50% MilliQ water
PFOA					50% Methanol+50% MilliQ water
PFNA					100% Methanol
PFUnA					100% Methanol
HFPO-DA					50% Methanol+50% MilliQ water
PFHxS					50% Methanol+50% MilliQ water
PFOS					100% Methanol
6:2 FTSA					100% Methanol
PFOSA					100% Methanol

(b)	C18 coating volume [nL]	Assay vials	Condition for extraction	Condition for desorption	Desorption solvent
PFBA	520	Screw vial with insert	24 h, 250rpm, 37℃	2h, 250rpm, 37℃	50% Methanol+50% MilliQ water
PFHxA	520	Screw vial with insert	24 h, 250rpm, 37℃	2h, 250rpm, 37℃	50% Methanol+50% MilliQ water
PFHpA	520	Screw vial with insert	24 h, 250rpm, 37℃	2h, 250rpm, 37℃	50% Methanol+50% MilliQ water
PFOA	520	Screw vial with insert	24 h, 250rpm, 37℃	2h, 250rpm, 37℃	50% Methanol+50% MilliQ water
PFNA	173	Crimp vial with insert	24 h, 1200rpm, 37℃	2h, 1200rpm, 37℃	100% Methanol
PFUnA	173	Crimp vial with insert	24 h, 1200rpm, 37℃	2h, 1200rpm, 37℃	100% Methanol
HFPO-DA	520	Screw vial with insert	24 h, 250rpm, 37℃	2h, 250rpm, 37℃	50% Methanol+50% MilliQ water
PFHxS	520	Screw vial with insert	24 h, 250rpm, 37℃	2h, 250rpm, 37℃	50% Methanol+50% MilliQ water
PFOS	173	Crimp vial with insert	24 h, 1200rpm, 37℃	2h, 1200rpm, 37℃	100% Methanol
6:2 FTSA	520	Screw vial with insert	24 h, 250rpm, 37℃	2h, 250rpm, 37℃	100% Methanol
PFOSA	173	Crimp vial with insert	24 h, 1200rpm, 37℃	2h, 1200rpm, 37℃	100% Methanol

Text S3. Experimental procedure of measuring cell binding assay of PFAS.

Step 1: Cells were detached with trypsin and collected as a pellet after centrifugation. Four cell pellets (HEK293H, MCF7, H4lle and SH-SY5Y) were resuspended with phosphate buffered saline (PBS) at a density of 10^7 cells/mL. Cell suspension was transferred to a 5 mL plastic vial (0030119401, Eppendorf) and homogenized by ultrasonic shattering (Sonoplus 2070, Germany) in an ice-water bath. The cell homogenate was diluted with PBS to a density of 2.5×10^6 cells/mL. PFAS stock solutions were diluted with PBS.

Step 2: 100µL cell homogenate and 100µL PFAS solution were added and vortexed in a 1.5mL HPLC vial with insert (7648146, 765116, Labsolute). Cell homogenates in each sample contained approximately 1.25×10^6 cells. 100µL PFAS solution and 100µL PBS were mixed and were used for the measurements of total molar amount (n_{tot}), which was used to calculate the total concentration (C_{tot}) in the cell homogenate as listed in Table S2.

Step3: Samples of PFBA, PFHxA, PFHpA, PFOA, HFPO-DA, PFHxS and 6:2 FTSA were prepared in screw vials with insert and 520 nL C18-coated fibers were used. Samples of PFNA, PFUnA, PFOS and PFOSA were prepared in crimp vials with insert and 173 nL C18-coated fibers were used. The experimental conditions are listed in Table S3b. These samples were used to derive the distribution ratios between cell and water ($D_{cell/w}$) as Qin et al.[2]

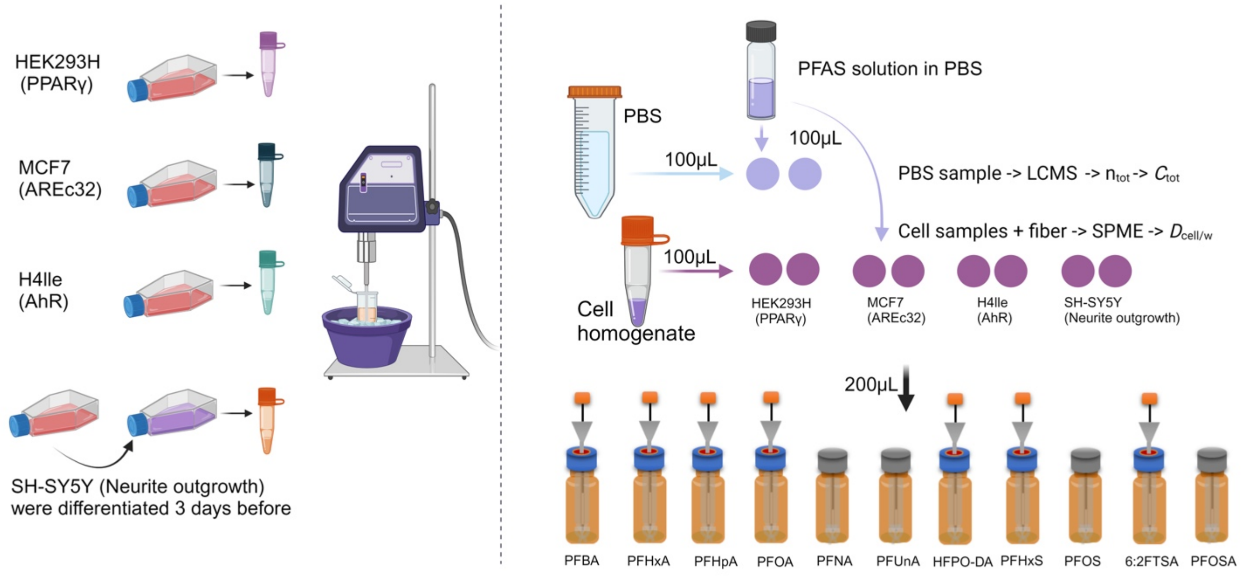

Figure S3. Experimental workflow of C18-SPME used in cell binding assays to measure distribution ratio of PFAS between cell and water ($D_{cell/w}$).

Text S4. Experimental procedure of structural protein binding assay of PFAS.

Step 1: Structural protein is not dissolved in PBS but a homogenous suspension at density of 100 mg/mL can be obtained after high-speed vortex (3 × 3 min). The pH value of suspension was adjusted gradually to 7.4 with sodium hydroxide. 11 PFAS solution were prepared in PBS individually.

Step 2: 500 μL PFAS solution and 500 μL structural protein were mixed in 1.5 mL vials (7654554, 7663230, Labsolute). 500 μL PFAS solution and 500 μL PBS were mixed and were used for the measurements of total molar amount (n_{tot}), which were used to calculate the total concentration (C_{tot}) in the protein suspension as listed in Table S2.

Step 3: Samples of PFBA, PFHxA, PFHpA, PFOA, HFPO-DA, PFHxS and 6:2 FTSA were prepared in screw vials and 520 nL C18-coated fibers were used. Samples of PFNA, PFUnA, PFOS and PFOSA were prepared in crimp vials and 173 nL C18-coated fibers were used. The experimental conditions are listed in Table S3b. These samples were used to derive the distribution ratios between structural protein and water ($D_{\mathrm{SP/w}}$).

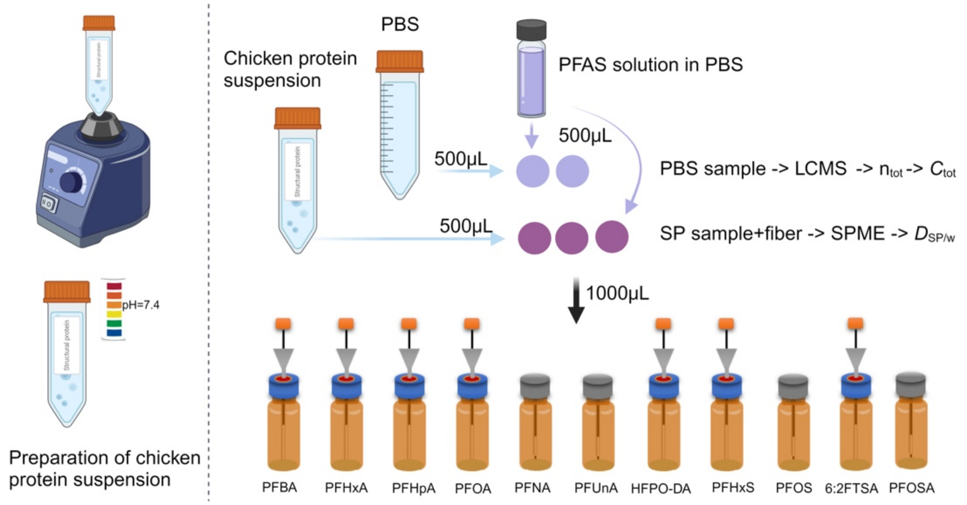

Figure S4. Experimental workflow of C18-SPME used in structural protein binding assays to measure distribution ratio of PFAS between structural protein and water ($D_{\mathrm{SP/w}}$).

Text S5. Experimental procedure of high throughput screening of PFAS in 384-well plates.

For the neurotoxicity assay, SH-SY5Y cells were differentiated with all-trans retinoic acid (R2625, Sigma-Aldrich) three days in advance. The following operations for four cell-based bioassays are similar and completed in three days. On the first day, cells were seeded in 384-well plate by a MultiFlo Dispenser (Biotek,Vermont, USA). On the second day, PFAS dosing. PFAS stock solution were prepared with methanol. Defined volumes of PFAS methanolic stock solutions were transferred to dosing vials (2214340, Labsolute) and blown down with nitrogen. PFAS were dissolved again in dosing medium. Then, serial concentrations of PFAS in dosing medium were prepared and dosed to cells by Hamilton Star Robot (Bonaduz, Switzerland). Cell plates were imaged by IncuCyte S3 at the start of exposure.

On the third day after 24 h of PFAS exposure, for the three reporter gene cell lines, the cytotoxicity was also analyzed by comparing confluency of the cells before and after 24 h of exposure. In case of the PPARγ-GeneBLAzer, the production of the β-lactamase reporter protein was measured with ToxBLAzer™ DualScreen Kit (Invitrogen™ K1138). The fluorescence at excitation of 409 nm and emission of 460 nm for blue light and 530 nm for green light were read with an Infinite® M1000 plate reader (Tecan, USA). The reporter protein luciferase of AREc32 and AhR-CALUX was quantified by bioluminescence with substrates prepared with D-luciferin (ABD-12506, AAT Bioquest). For SH-SY5Y cell lines, neurite length of differentiated SH-SY5Y cells was quantified by phase-contrast imaging using an IncuCyte S3. Then, Nuclear Green LCS1 (ab138904, Abcam) and propidium iodide (81845, Sigma Aldrich) were used to stain the total cells and death cells for one hour.

The inhibitory concentrations triggering 10% cytotoxicity (IC_{10}) and 10% of maximum effect (EC_{10}) were calculated by 10% divided a slope of a linear concentration response curve (CRC).[3] For antagonism of PPARγ, the suppression ratio SPR of 20% is often used and EC_{SPR20} were calculated by 20% divided a slope of a linear CRC.[2] For AREc32, the concentration causing an induction ratio of 1.5, $EC_{IR1.5}$, was derived from a linear CRC through the intercept IR 1.[4]

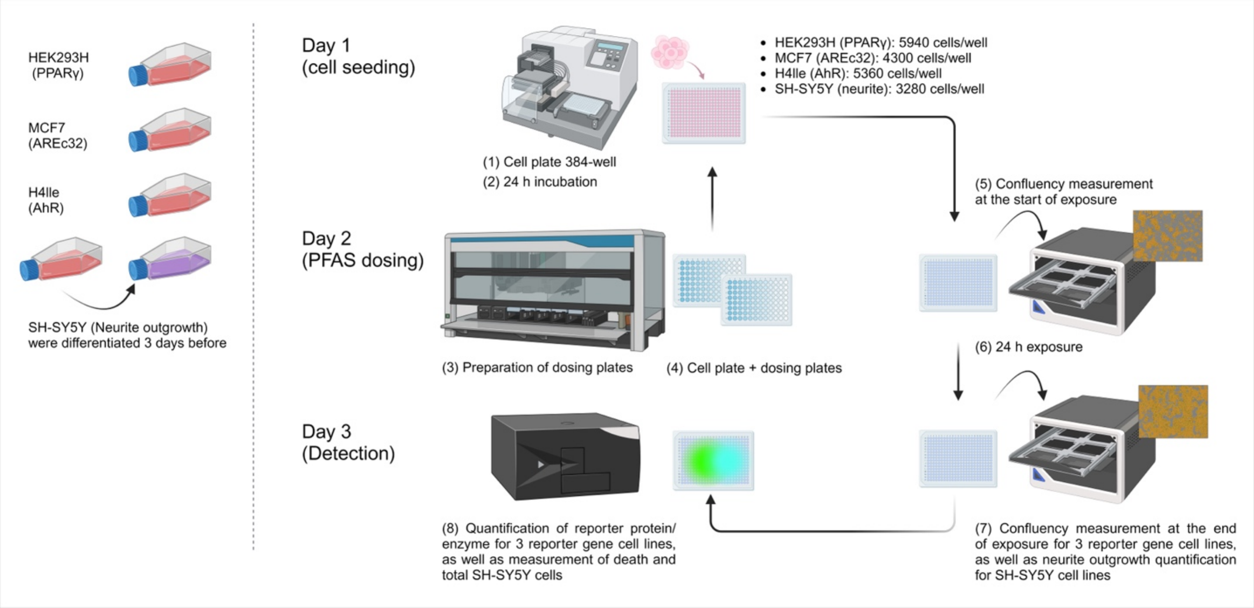

Figure S5. Experimental workflow of high throughput screening of PFAS in 384-well plates.

Table S4. Information on (a) cells and (b) medium used for the four cell-based HTS bioassays.

(a)	Cell information			Reference compound		
	Cell line	Source	Cell number	Targets	Chemical name	DTXSID
PPARγ reporter gene assay	HEK293H	Thermo Fisher Scientific	5940	peroxisome proliferator-activated receptor γ	Rosiglitazone	DTXSID7037131
AREc32 reporter gene assay	MCF7	Cancer research UK	4300	antioxidant response element	tert-Butyl hydroquinone	DTXSID6020220
AhR CALUX reporter gene assay	H4lle	UC Davis, USA	5360	aryl hydrocarbon receptor	2,3,7,8Tetrachloro-dibenzo-p-dioxin	DTXSID2021315
Neurotoxicity based on neurite outgrowth	SH-SY5Y	Sigma Aldrich	3280	neurotoxicity assay that quantifies neurite length	Narciclasine	DTXSID70183677

(b)	Assay medium				Plates
	Medium base	Source	Serum/substrate	Source	384-well plate
PPARγ reporter gene assay	98% OptiMEM	Cat.11058021, GIBCO	2% charcoal stripping (cs) fetal bovine serum (FBS)	12676029,GIBCO	354663, Corning
AREc32 reporter gene assay	90% DMEM	Cat.31966021, GIBCO	10% fetal bovine serum	10099141,GIBCO	3765, Corning
AhR CALUX reporter gene assay	90% DMEM	Cat.31966021, GIBCO	10% fetal bovine serum	10099141,GIBCO	356660, Corning
Neurotoxicity based on neurite outgrowth	Neurobasal medium	Cat.12348017, GIBCO	2% B-27 Supplement and 2% GlutaMAX Supplement	17504044,35050061,GIBCO	356667, Corning

Table S5. Maximum concentrations of 24 PFAS in four cell-based HTS bioassays.

[mol/L]	Chemicals	PPARγ reporter gene assay	AREc32 reporter gene assay	AhR CALUX reporter gene assay	Neurotoxicity based on neurite outgrowth
1	PFPrA	1.80×10^{-2}	7.18×10^{-2}	7.18×10^{-2}	2.74×10^{-2}
2	PFBA	3.88×10^{-3}	7.78×10^{-3}	7.77×10^{-3}	1.07E-02
3	PFPeA	3.73×10^{-3}	7.46×10^{-3}	7.46×10^{-3}	4.52×10^{-3}
4	PFHxA	2.70×10^{-3}	2.71×10^{-3}	2.70×10^{-3}	3.07×10^{-3}
5	PFHpA	1.30×10^{-3}	1.30×10^{-3}	1.30×10^{-3}	1.85×10^{-3}
6	PFOA	1.30×10^{-3}	1.37×10^{-3}	1.37×10^{-3}	1.23×10^{-3}
7	PFNA	6.82×10^{-4}	6.85×10^{-4}	6.82×10^{-4}	1.07×10^{-3}
8	PFUnA	1.45×10^{-4}	2.40×10^{-3}	5.81×10^{-4}	2.45×10^{-4}
9	PFDoDA	1.65×10^{-4}	1.66×10^{-3}	3.31×10^{-4}	1.05×10^{-4}
10	PFOdA	1.47×10^{-3}	8.82×10^{-3}	8.82×10^{-3}	2.16×10^{-3}
11	HFPO-DA	4.79×10^{-3}	4.79×10^{-3}	4.79×10^{-3}	2.65×10^{-3}
12	TfOH	1.40×10^{-2}	2.81E-02	2.81×10^{-2}	3.13×10^{-2}
13	PFHxS	1.99×10^{-4}	3.99×10^{-3}	7.96×10^{-4}	5.11×10^{-4}
14	PFOS	2.27×10^{-4}	1.95×10^{-3}	2.85×10^{-4}	2.29×10^{-4}
15	6:2 FTSA	6.65×10^{-4}	1.33×10^{-3}	1.33×10^{-3}	1.91×10^{-3}
16	PFOSA	9.28×10^{-5}	6.82×10^{-4}	1.86×10^{-4}	4.12×10^{-5}
17	nEt-PFOSA	8.09×10^{-5}	1.62×10^{-4}	1.62×10^{-4}	4.33×10^{-5}
18	nEt-PFOSE	7.62×10^{-5}	1.52×10^{-4}	1.52×10^{-4}	3.90×10^{-5}
19	6:2 FTOH	9.53×10^{-4}	1.91×10^{-3}	1.91×10^{-3}	1.66×10^{-3}
20	8:2 FTOH	4.18×10^{-4}	8.36×10^{-4}	8.36×10^{-4}	5.47×10^{-4}
21	10:2 FTOH	2.02×10^{-4}	2.03×10^{-4}	2.02×10^{-4}	1.91×10^{-4}
22	Hexaflumuron	1.91×10^{-4}	1.92×10^{-4}	1.91×10^{-4}	4.87×10^{-5}
23	Lufenuron	1.01×10^{-4}	2.02×10^{-4}	1.01×10^{-4}	9.51×10^{-5}
24	Flubendiamide	1.38×10^{-4}	2.76×10^{-4}	1.38×10^{-4}	8.39×10^{-5}

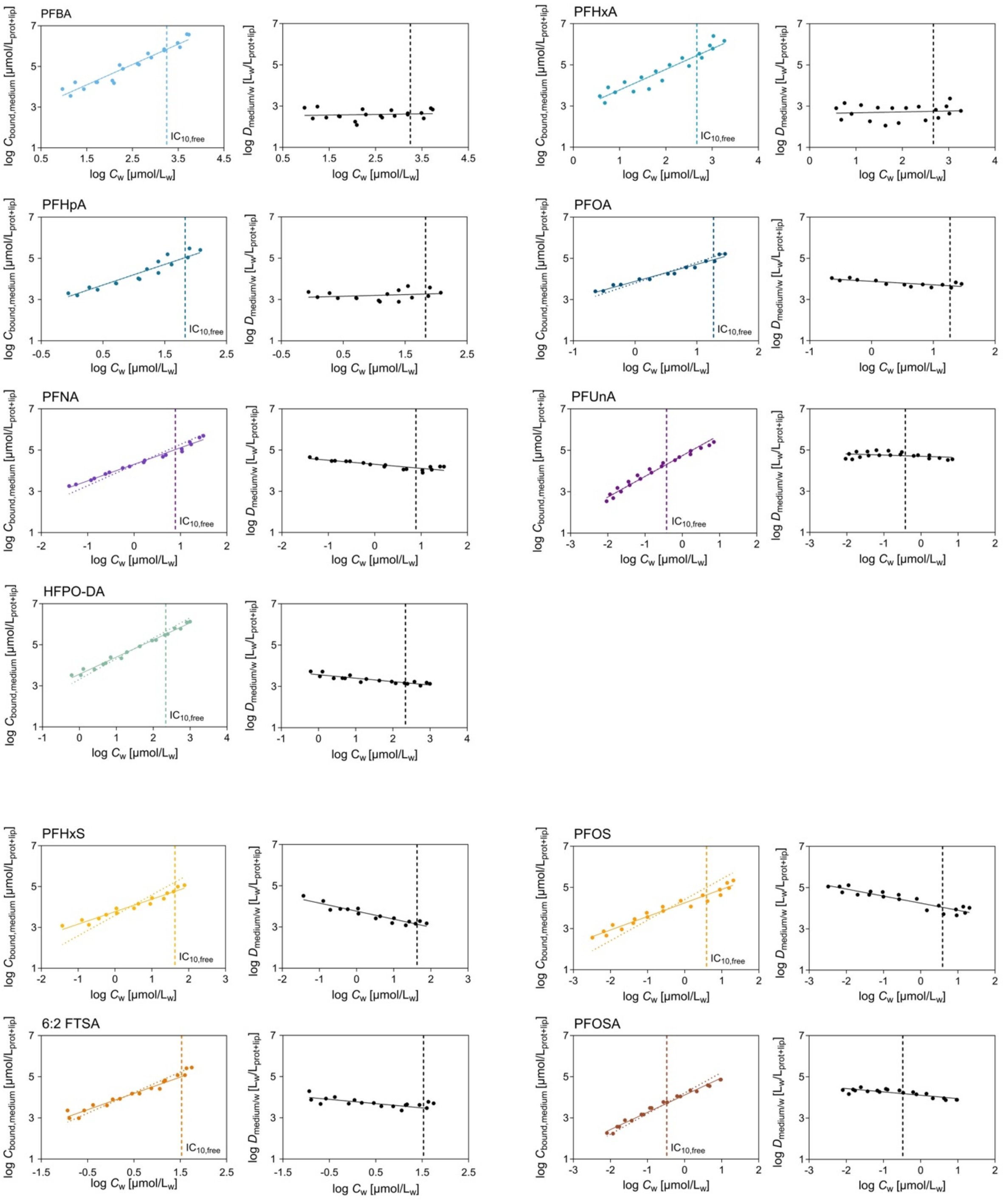

Figure S6. Medium binding isotherms of 11 PFAS. These isotherms were analyzed with a Freundlich-type model[2] to derive regression equations between log $D_{\mathrm{medium/w}}$ against log C_{w} as shown in Table S6. Concentration unit of PFAS in the water phase (C_{w}) is micromolar [μmol/L$_{\mathrm{w}}$], where L$_{\mathrm{w}}$ is liter of water. IC$_{10,\mathrm{free}}$ related to cytotoxicity were derived from PPARγ-GeneBLAzer reporter gene assays as shown in Figure S7.

Table S6. Distribution ratios between medium and water ($D_{medium/w}$) and distribution ratios between BSA and water ($D_{BSA/w}$) of 11 PFAS. Regression equations between log $D_{medium/w}$ and $D_{BSA/w}$ against log C_w were derived using a Freundlich-type model.[2] The concentration unit of PFAS in the water phase (C_w) is micromolar [μmol/L].

	**log $D_{medium/w}$ [$L_w/L_{prot+lip}$]**		**log $D_{BSA/w}$ [L_w/L_{prot}]**	
	**Equation**	**R^2**	**Equation**	**R^2**
**PFBA**	2.60	0.94	$\log D_{BSA/w} = -0.298 \log C_w + 2.91$	0.60
**PFHxA**	2.78	0.90	$\log D_{BSA/w} = -0.271 \log C_w + 3.43$	0.88
**PFHpA**	3.21	0.91	$\log D_{BSA/w} = -0.305 \log C_w + 3.95$	0.81
**PFOA**	$\log D_{medium/w} = -0.172 \log C_w + 3.88$	0.60	$\log D_{BSA/w} = -0.314 \log C_w + 4.38$	0.93
**PFNA**	$\log D_{medium/w} = -0.197 \log C_w + 4.31$	0.67	$\log D_{BSA/w} = -0.147 \log C_w + 4.52$	0.61
**PFUnA**	4.74	0.97	4.75	0.96
**HFPO-DA**	$\log D_{medium/w} = -0.167 \log C_w + 3.57$	0.79	$\log D_{BSA/w} = -0.493 \log C_w + 3.44$	0.86
**PFHxS**	$\log D_{medium/w} = -0.389 \log C_w + 3.77$	0.89	$\log D_{BSA/w} = -0.472 \log C_w + 4.28$	0.92
**PFOS**	$\log D_{medium/w} = -0.343 \log C_w + 4.25$	0.86	$\log D_{BSA/w} = -0.379 \log C_w + 4.74$	0.92
**6:2 FTSA**	$\log D_{medium/w} = -0.207 \log C_w + 3.79$	0.66	$\log D_{BSA/w} = -0.092 \log C_w + 3.86$	0.54
**PFOSA**	$\log D_{medium/w} = -0.162 \log C_w + 4.11$	0.70	$\log D_{BSA/w} = -0.105 \log C_w + 4.28$	0.50

Table S7. Volume fractions of protein and lipid in medium used in the four cell-based HTS bioassays. The generic medium was defined for a common experimental condition using 10% FBS.

**Assay name**	**Assay medium**	**$Vf_{protein,medium}$ [mL/L]**	**$Vf_{lipid,medium}$ [mL/L]**	**$Vf_{w,medium}$ [L/L]**	**$V_{total,medium}$ [L]**
**PPARγ reporter gene assay**	98% OptiMEM+2% csFBS	0.94	1.47×10^{-2}	0.999	4.00×10^{-5}
**AREc32 reporter gene assay**	90% DMEM+10% FBS	3.02	7.44×10^{-2}	0.997	4.00×10^{-5}
**AhR reporter gene assay**	90% DMEM+10% FBS	3.02	7.44×10^{-2}	0.997	4.00×10^{-5}
**Neurotoxicity based on neurite outgrowth**	Neurobasal medium+2% B-27 Supplement+2% GlutaMAX Supplement	2.38	3.89×10^{-3}	0.997	4.00×10^{-5}
**Generic medium**	10% FBS	**3.0**	$\mathbf{7\times10^{-2}}$	**0.997**	$\mathbf{4.00\times10^{-5}}$

Table S8. Volume fractions of protein and lipid in cells used in the four cell-based HTS bioassays. The generic cell was defined based on average values measured from four cell lines.

**Assay name**	**Cell line**	**$C_{protein}$ [mL/10^6 cell]**	**C_{lipid} [mL/10^6 cell]**	**$V_{total,cell}$ [mL/10^6 cell]**	**cell number in 384-well plate**	**$Vf_{protein,cell}$ [mL/L]**	**$Vf_{lipid,cell}$ [mL/L]**	**$Vf_{w,cell}$ [L/L]**	**$V_{tot, cell}$ [L]**
**PPARγ reporter gene assay**	HEK293H	9.73×10^{-5}	1.53×10^{-5}	2.87×10^{-3}	5940	33.9	5.3	0.961	1.71×10^{-8}
**AREc32 reporter gene assay**	MCF7	1.87×10^{-4}	4.09×10^{-5}	1.00×10^{-2}	4300	18.7	4.1	0.977	4.30×10^{-8}
**AhR reporter gene assay**	H4lle	9.00×10^{-5}	1.46×10^{-5}	3.53×10^{-3}	5360	25.5	4.1	0.970	1.89×10^{-8}
**Neurotoxicity**	SH-SY5Y	6.67×10^{-5}	9.58×10^{-5}	1.96×10^{-3}	3280	34.0	4.9	0.961	6.43×10^{-9}
**Generic cells**						**30**	**5**	**0.965**	$\mathbf{3.00\times10^{-8}}$

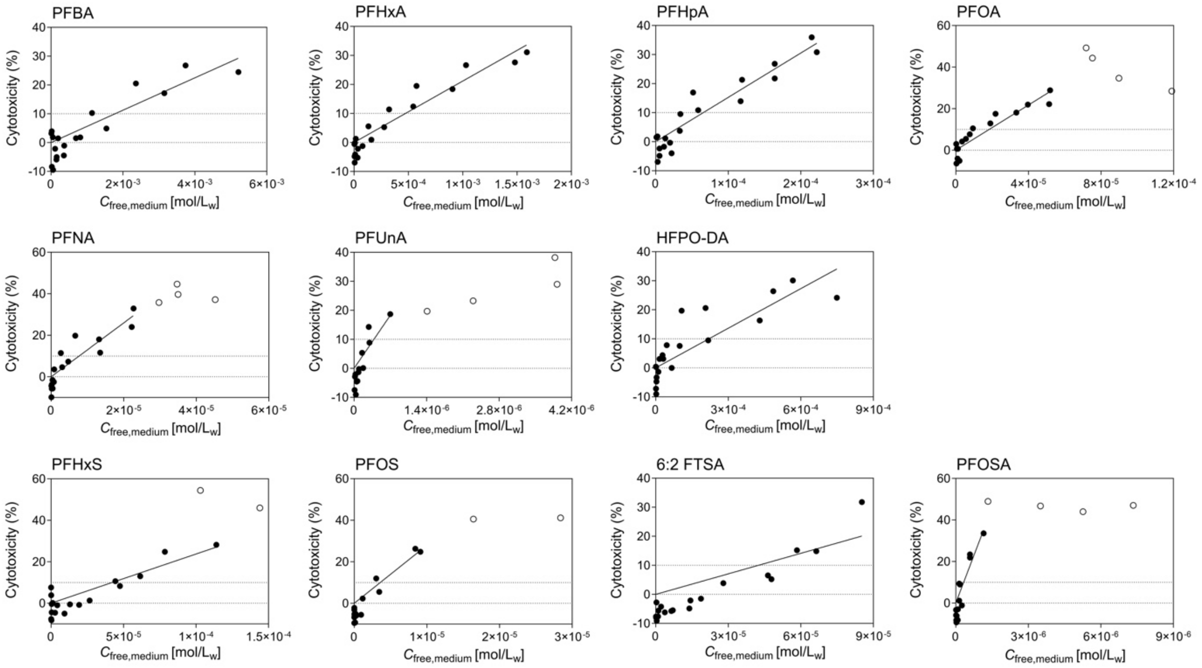

Figure S7. Cytotoxicity of 11 PFAS in the PPARγ-GeneBLAzer reporter gene assay. Inhibitory concentration $IC_{10,free}$ were derived from the concentration-response curves at 10% cytotoxicity with measured free concentrations in the medium $C_{free,medium}$. Values of cytotoxicity more than 40% were excluded from the linear fitting (hollow circle).

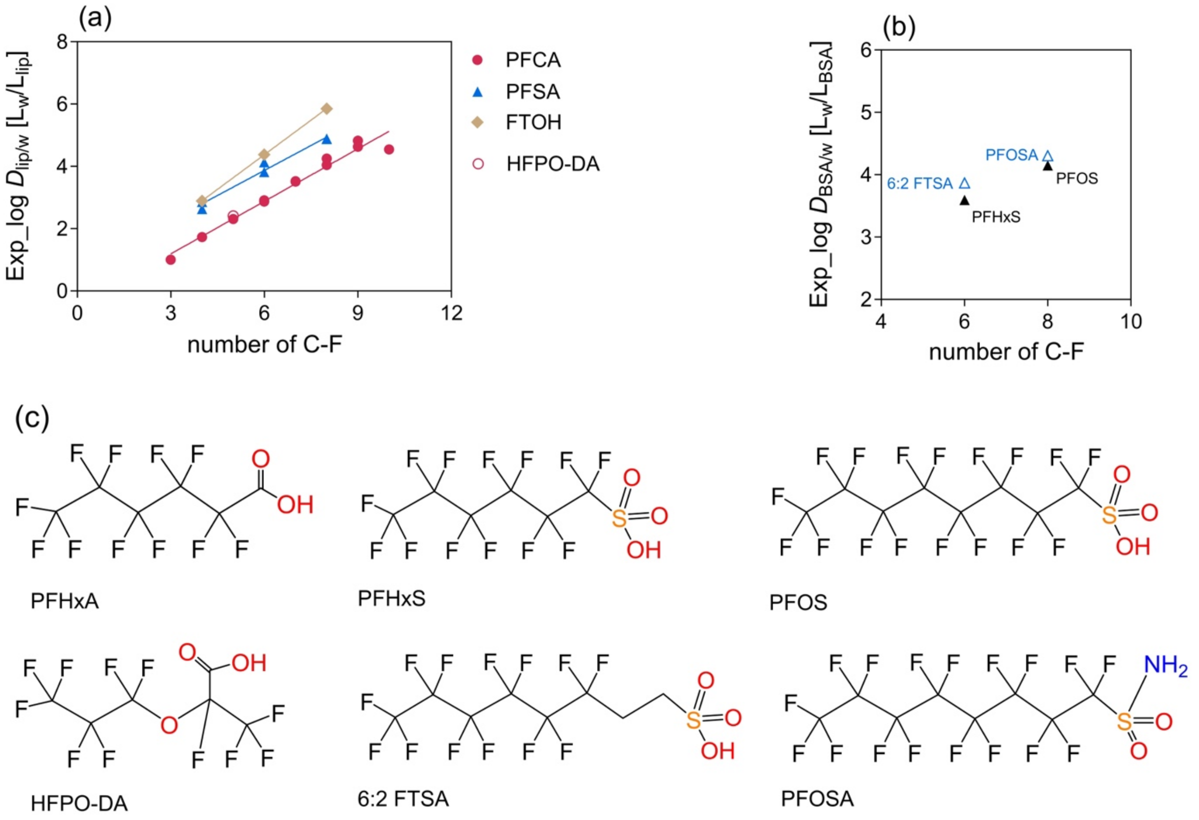

Figure S8. Information of lipid and protein bindings and PFAS structures. **(a)** Linear relationship of distribution ratios between liposomes and water (log $D_{lip/w}$) against the number (N) of perfluorinated carbons. **(b)** Distribution ratios between bovine serum albumin and water (log $D_{BSA/w}$) of perfluorohexanesulfonate (PFHxS), perfluorooctanesulfonate (PFOS), 6:2 fluorotelomer sulfonic acid (6:2FTSA) and perfluorooctanesulfonamide (PFOSA). **(c)** Structures of perfluorohexanoic acid (PFHxA), perfluoro-2-methyl-3-oxahexanoic acid (HFPO-DA), PFHxS, 6:2 FTSA, PFOS and PFOSA.

$D_{lip/w}$ of perfluoroalkyl carboxylic acid (PFCA), perfluoroalkyl sulfonic acid (PFSA) and fluorotelomer alcohol (FTOH) were measured by Droge et al.,[5] Ebert et al.[6] and Endo et al.[7] Linear regressions of log $D_{lip/w}$ against the number (N) of perfluorinated carbons were fitted for PFCA (magenta circles in Figure S8a, Eq. S9), PFSA (blue triangles in Figure S8a, Eq. S10) and FTOH (gold diamond in Figure S8a, Eq. S11).

PFCA: log $D_{lip/w}$=0.562×N-0.494 (R^2=0.969) (S9)

PFSA: log $D_{lip/w}$=0.535×N+0.658 (R^2=0.977) (S10)

FTOH: log $D_{lip/w}$=0.740×N-0.067 (R^2=0.999) (S11)

$D_{lip/w}$ of HFPO-DA (empty magenta circle in Figure S8a) were overlaying the regression of PFCA even though the structure is different from PFHxA with five C-F and an ether group. $D_{BSA/w}$ of PFOSA is close

to that of PFOS because of their similar structures with eight perfluorinated carbon. Although there are eight carbons (six C-F and two C-H) in the structure of 6:2 FTSA, its $D_{BSA/w}$ is closer to PFHxS with six C-F. Therefore, the prediction of $D_{lip/w}$ of PFOSA was done with N=8 and of 6:2 FTSA with N=6 by Eq. S10.

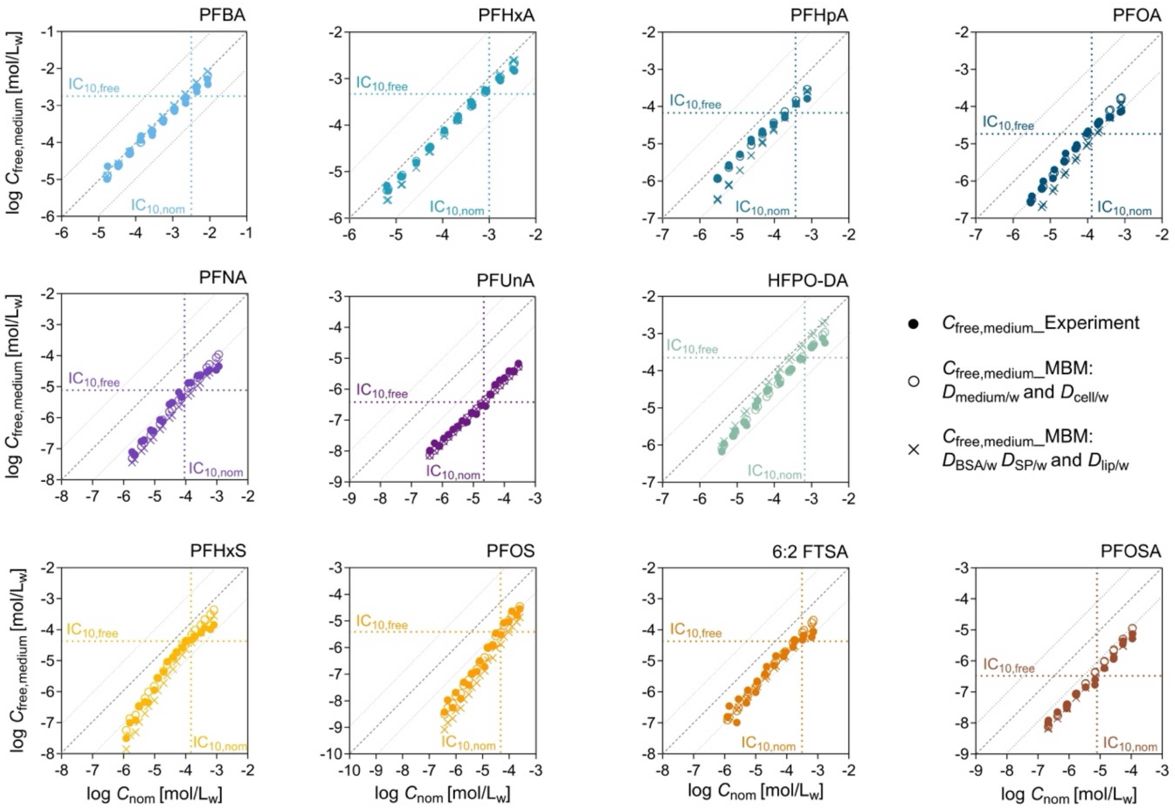

Figure S9. Relationship between nominal (C_{nom}) and measured free ($C_{free,medium}$) concentrations of PFAS in the PPARγ-GeneBLAzer assay. $C_{free,medium}$ were predicted concentration-dependently by the mass balance model (MBM) from C_{nom} with distribution ratio between medium and water ($D_{medium/w}$) and cell and water ($D_{cell/w}$) measured in this study (Eq. 3), or with distribution ratio between BSA and water ($D_{BSA/w}$), between structural protein and water ($D_{SP/w}$) and liposome and water ($D_{lip/w}$) (Eq. 6). Inhibitory concentration of 10% cytotoxicity $IC_{10,nom}$ or $IC_{10,free}$ were derived from concentration-response curves at 10% cytotoxicity with C_{nom} or measured $C_{free,medium}$.

Table S9. Free and nominal concentrations of 11 PFAS related to baseline toxicity. $IC_{10,free,baseline}$ were calculated with Eq.8 in main manuscript and $IC_{10,nom,baseline}$ were predicted by the mass balance model (MBM, Eqs. 3 or 6).

Abbreviation	$IC_{10,free,baseline}$ [mol/L] (Eq. 8)	$IC_{10,nom,baseline}$ [mol/L] (MBM, Eq. 3)	$IC_{10,nom,baseline}$ [mol/L] (MBM, Eq. 6)
**PFBA**	6.90×10^{-3}	9.51×10^{-3}	7.52×10^{-3}
**PFHxA**	3.30×10^{-4}	5.33×10^{-4}	4.85×10^{-4}
**PFHpA**	8.49×10^{-5}	2.21×10^{-4}	3.22×10^{-4}
**PFOA**	2.08×10^{-5}	1.14×10^{-4}	1.70×10^{-4}
**PFNA**	3.88×10^{-6}	6.22×10^{-5}	1.53×10^{-4}
**PFUnA**	1.99×10^{-6}	1.07×10^{-4}	1.09×10^{-4}
**HFPO-DA**	2.68×10^{-4}	6.45×10^{-4}	3.08×10^{-4}
**PFHxS**	5.12×10^{-6}	2.06×10^{-5}	2.44×10^{-5}
**PFOS**	8.89×10^{-7}	1.67×10^{-5}	1.45×10^{-5}
**6:2 FTSA**	9.35×10^{-6}	4.51×10^{-5}	7.58×10^{-5}
**PFOSA**	7.96×10^{-7}	1.10×10^{-5}	1.76×10^{-5}

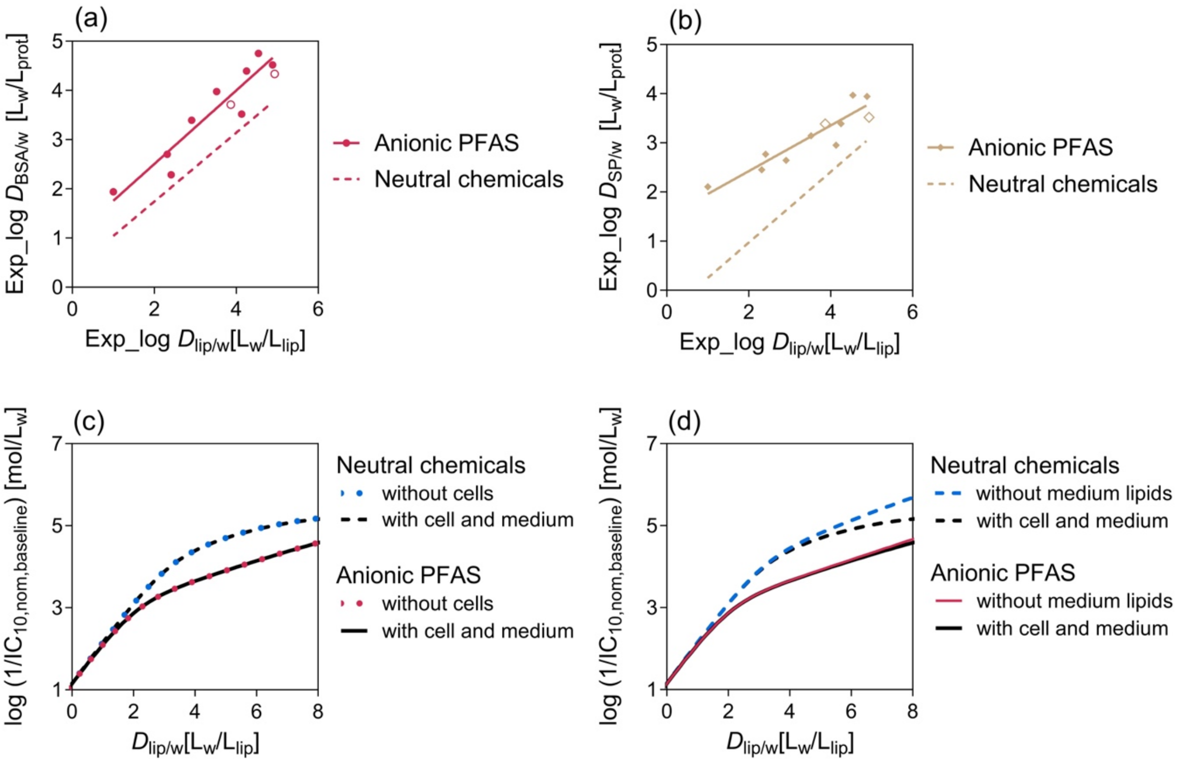

Figure S10. Relationships of protein and lipid binding and distribution of medium and cells in the baseline toxicity prediction models. **(a)** Linear relationship of measured distribution ratios between bovine serum albumin and water ($D_{BSA/w}$, Table 1) against measured distribution ratios between liposome and water ($D_{lip/w}$, Table 1) for anionic PFAS (Eq. 14) and neutral organic chemicals from literature[8] (Eq. 16). The empty symbols are 6:2 FTSA and PFOSA, which were not included in the regression (Eq. 14) because their $D_{lip/w}$ were predicted. **(b)** Linear relationship of distribution ratios between structural protein and water ($D_{SP/w}$, Table 1) against $D_{lip/w}$ for anionic PFAS (Eq. 15) and neutral organic chemicals from literature[9] (Eq. 17). 6:2 FTSA and PFOSA (empty symbols) were not included in the regression (Eq. 15). **(c)** Contributions of cell binding to the baseline toxicity prediction with generic cell models. Cells were excluded and only medium was considered in the model for anionic PFAS (Eq.19) and model for neutral chemicals (Eq.20). (**d**) Contributions of distribution to medium lipids to the baseline toxicity prediction models.

Table S10. Distribution ratios between cell and water ($D_{cell/w}$) of 11 PFAS with four cell lines.

**log $D_{cell/w}$ [$L_w/L_{prot+lip}$]**	**HEK293H (PPARγ)**	**MCF7 (AREc32)**	**H4lle (AhR)**	**SH-SYSY (neurotoxicity)**	**Average (±SE)**
**PFBA**	2.40	2.71	2.54	2.02	2.42±0.15
**PFHxA**	3.45	3.46	3.52	3.83	3.57±0.09
**PFHpA**	3.69	3.49	3.65	3.69	3.63±0.05
**PFOA**	3.94	3.97	3.76	3.78	3.86±0.06
**PFNA**	3.74	3.22	3.67	3.61	3.56±0.11
**PFUnA**	3.78	3.12	3.89	3.28	3.52±0.19
**HFPO-DA**	3.09	3.16	3.07	2.91	3.06±0.05
**PFHxS**	3.56	3.33	3.59	3.71	3.55±0.08
**PFOS**	4.32	5.06	4.52	4.68	4.64±0.16
**6:2 FTSA**	3.90	3.87	4.13	4.31	4.05±0.10
**PFOSA**	3.81	3.89	3.97	3.92	3.90±0.03

Table S11. Chemical information of anionic and neutral PFAS. 24 PFAS[a] were used in this study and 16 PFAS[b] were from Evans et al.[10] Distribution ratios of PFAS between liposome and water ($D_{lip/w}$) were from literature or predicted with Eqs.S9, S10 and S11 as shown in Figure S8. $IC_{10,free,baseline}$ were calculated with Eq.8 in main manuscript and $IC_{10,nom,baseline}$ were predicted by the baseline toxicity generic models (Eq.11, Table 2).

	**DTXSID**	**Abbreviation**	**log $D_{lip/w}$ [L_w/L_{lip}]**	**Source**	**$IC_{10,free,baseline}$ [mol/L] (Eq. 8)**	**$IC_{10,nom,baseline}$ [mol/L] (Eq. 11)**
**Anionic at pH=7.4 (20)**						
**1**	DTXSID8059970	PFPrA [a]	0.63	Predicted with Eq. S9	1.62×10^{-2}	1.60×10^{-2}
**2**	DTXSID4059916	PFBA [a]	1.00	Droge et al. 2019[5]	6.90×10^{-3}	8.06×10^{-3}
**3**	DTXSID6062599	PFPeA [a]	1.75	Predicted with Eq. S9	1.22×10^{-3}	2.42×10^{-3}
**4**	DTXSID3031862	PFHxA [a,b]	2.32	Ebert et al. 2020[6]	3.30×10^{-4}	1.13×10^{-3}
**5**	DTXSID1037303	PFHpA [a]	2.91	Ebert et al. 2020[6]	8.49×10^{-5}	5.73×10^{-4}
**6**	DTXSID8031865	PFOA [a,b]	3.52	Ebert et al. 2020[6]	2.08×10^{-5}	3.14×10^{-4}
**7**	DTXSID8031863	PFNA [a,b]	4.25	Ebert et al. 2020[6]	3.88×10^{-6}	1.72×10^{-4}
**8**	DTXSID3031860	PFDA [b]	4.64	Ebert et al. 2020[6]	1.58×10^{-6}	1.30×10^{-4}
**9**	DTXSID8047553	PFUnA [a]	4.54	Ebert et al. 2020[6]	1.99×10^{-6}	1.39×10^{-4}
**10**	DTXSID8031861	PFDoDA [a]	5.69	Predicted with Eq. S9	1.42×10^{-7}	6.98×10^{-5}
**11**	DTXSID00369821	PFOdA [a]	2.88	Predicted with Eq. S9	9.14×10^{-5}	5.93×10^{-4}
**12**	DTXSID50904660	PFMOAA [b]	0.63	Predicted with Eq. S9	1.62×10^{-2}	1.60×10^{-2}
**13**	DTXSID40108559	HFPO-DA-AS [b]	2.32	Predicted with Eq. S9	3.33×10^{-4}	1.14×10^{-3}
**14**	DTXSID70880215	HFPO-DA [a,b]	2.41	Ebert et al. 2020[6]	2.68×10^{-4}	1.01×10^{-3}
**15**	DTXSID2044397	TfOH [a]	1.19	Predicted with Eq. S10	4.42×10^{-3}	5.79×10^{-3}
**16**	DTXSID5030030	PFBS [b]	3.51	Ebert et al. 2020[6]	2.13×10^{-5}	3.17×10^{-4}
**17**	DTXSID3037709	PFHxS [a,b]	4.13	Ebert et al. 2020[6]	5.12×10^{-6}	1.88×10^{-4}
**18**	DTXSID8037706	PFOS [a,b]	4.89	Ebert et al. 2020[6]	8.89×10^{-7}	1.10×10^{-4}
**19**	DTXSID6067331	6:2 FTSA [a]	3.87	Predicted with Eq. S10	9.35×10^{-6}	2.32×10^{-4}
**20**	DTXSID10892352	NBP2 [b]	4.40	Predicted with Eq. S10	2.73×10^{-6}	1.53×10^{-4}
**Partially charged at pH=7.4 (4)**						
**21**	DTXSID3038939	PFOSA [a,b]	4.94	Predicted with Eq. S10	7.96×10^{-7}	2.63×10^{-5} (92% neutral)
**22**	DTXSID3032620	Hexaflumuron [a]	4.58	From COSMOtherm	1.83×10^{-6}	3.47×10^{-5} (98% neutral)
**23**	DTXSID5034357	Lufenuron [a]	5.21	From COSMOtherm	4.29×10^{-7}	2.18×10^{-5} (98% neutral)
**24**	DTXSID4047672	Flubendiamide [a]	3.28	From COSMOtherm	3.61×10^{-5}	1.21×10^{-4} (98% neutral)
**Neutral at pH=7.4 (6)**						
**25**	DTXSID1032646	nEt-PFOSA [a,b]	5.37	From COSMOtherm	2.94×10^{-7}	1.95×10^{-5}
**26**	DTXSID6027426	nEt-PFOSE [a]	5.28	From COSMOtherm	3.65×10^{-7}	2.08×10^{-5}
**27**	DTXSID1062122	4:2 FTOH [b]	2.89	Endo et al. 2014[7]	8.89×10^{-5}	1.95×10^{-4}
**28**	DTXSID5044572	6:2 FTOH [a,b]	4.38	Endo et al. 2014[7]	2.88×10^{-6}	4.08×10^{-5}
**29**	DTXSID7029904	8:2 FTOH [a,b]	5.85	Endo et al. 2014[7]	9.75×10^{-8}	1.46×10^{-5}
**30**	DTXSID2029905	10:2 FTOH [a]	7.33	Predicted with Eq. S11	3.21×10^{-9}	7.33×10^{-6}

[a] 24 PFAS were used in this study.
[b] 16 PFAS were from Evans et al. 2022.[10]

Table S12. Cell responses of 24 PFAS in four cell-based HTS bioassays. $IC_{10,nom}$ refers to nominal concentrations in molar units (mol/L) and was derived from concentration-response curves (CRC) at 10% cytotoxicity. $EC_{10,nom}$ or $EC_{SPR10,nom}$ is the nominal effective concentration and derived from CRC at 10% agonistic effects on PPARγ, AREc32, AhR and neurotoxicity, or 20% antagonistic effects on PPARγ. Toxic ratio (TR, Eq. 12) and specificity ratio (SR, Eq. 13) of 24 PFAS were calculated with baseline toxicity prediction models for anionic PFAS (Eq. 18) and neutral chemicals (Eq. 19) in four cell-based HTS: PPARγ-GeneBLAzer, AREc32, AhR CALUX and neurotoxicity assays.

**Abbre-viation**	**Cytotoxicity**				**Cell endpoint response (SR)**				
	**PPARγ IC_{10} (TR)**	**AREc32 IC_{10} (TR)**	**AhR_ IC_{10} (TR)**	**Neurotoxicity_ IC_{10} (TR)**	**PPARγ EC_{10} (SR)**	**PPARγ_ EC_{SPR20} (SR)**	**AREc32 EC_{10} (SR)**	**AhR EC_{10} (SR)**	**Neurotoxicity_ EC_{10} (SR)**
**Anionic at pH=7.4 (n=15)**									
**PFPrA**	1.06×10^{-2} (1.07)	2.94×10^{-2} (0.54)	3.56×10^{-2} (0.45)	6.38×10^{-3} (2.45)	1.48×10^{-3} (7.62)	/	NA	1.61×10^{-2} (0.99)	NA
**PFBA**	3.14×10^{-3} (1.57)	6.06×10^{-3} (1.33)	5.09×10^{-3} (1.38)	1.95×10^{-3} (3.98)	5.81×10^{-4} (8.49)	/	NA	NA	1.32×10^{-3} (5.89)
**PFPeA**	NA	1.37×10^{-3} (1.76)	2.71×10^{-3} (0.89)	2.21×10^{-3} (1.01)	7.03×10^{-4} (1.61)	/	NA	NA	2.07×10^{-3} (1.08)
**PFHxA**	9.40×10^{-4} (0.47)	1.06×10^{-3} (1.06)	1.21×10^{-3} (0.93)	1.24×10^{-3} (0.81)	3.10×10^{-4} (1.44)	/	NA	NA	NA
**PFHpA**	3.57×10^{-4} (0.54)	5.17×10^{-4} (1.11)	3.22×10^{-4} (1.78)	1.03×10^{-3} (0.48)	NA	1.87×10^{-4} (1.03)	NA	NA	NA
**PFOA**	1.18×10^{-4} (0.77)	2.27×10^{-4} (1.38)	2.27×10^{-4} (1.38)	5.77×10^{-4} (0.45)	NA	1.47×10^{-5} (6.18)	NA	NA	NA
**PFNA**	1.30×10^{-4} (0.33)	2.66×10^{-4} (0.65)	1.86×10^{-4} (0.93)	3.33×10^{-4} (0.41)	NA	6.49×10^{-6} (6.55)	NA	NA	3.30×10^{-4} (0.41)
**PFUnA**	5.86×10^{-5} (0.56)	1.14×10^{-3} (0.12)	NA	NA	NA	5.04×10^{-5} (0.65)	NA	NA	NA
**PFDoDA**	NA	6.64×10^{-5} (1.06)	2.72×10^{-4} (0.26)	NA	NA	NA	NA	NA	NA
**PFOdA**	NA	4.20×10^{-3} (0.14)	1.77×10^{-3} (0.34)	NA	1.13×10^{-3} (0.18)	/	NA	NA	NA
**HFPO-DA**	1.31×10^{-3} (0.30)	4.12×10^{-4} (2.46)	1.64×10^{-3} (0.62)	1.15×10^{-3} (0.78)	1.33×10^{-5} (29.22)	9.16×10^{-4} (0.42)	NA	NA	NA
**TfOH**	NA	NA	1.30×10^{-2} (0.45)	3.47×10^{-3} (1.59)	7.53×10^{-3} (0.44)	/	NA	NA	NA
**PFHxS**	NA	2.39×10^{-4} (0.79)	4.30×10^{-4} (0.44)	4.53×10^{-4} (0.33)	NA	9.75×10^{-5} (0.49)	NA	NA	NA
**PFOS**	1.75×10^{-4} (0.14)	6.85×10^{-4} (0.16)	NA	NA	NA	2.39×10^{-5} (1.01)	NA	NA	NA
**6:2 FTSA**	NA	NA	NA	NA	NA	4.45×10^{-5} (1.40)	NA	NA	NA
**Partially charged at pH=7.4 (n=4)**									
**PFOSA**	1.40×10^{-5} (0.72)	7.13×10^{-5} (0.38)	3.46×10^{-5} (0.77)	NA	NA	7.29×10^{-6} (1.38)	NA	NA	NA
**Hexaflumuron**	NA	NA	6.71×10^{-5} (0.52)	NA	1.48×10^{-4} (0.10)	/	NA	NA	3.04×10^{-5} (0.68)
**Lufenuron**	NA	9.33×10^{-5} (0.24)	8.83×10^{-5} (0.25)	NA	7.36×10^{-6} (1.08)	/	NA	8.38×10^{-5} (0.26)	4.98×10^{-5} (0.23)
**Flubendiamide**	NA	NA	NA	NA	NA	1.20×10^{-4} (0.55)	NA	NA	NA
**Neutral at pH=7.4 (n=5)**									
**nEt-PFOSA**	NA	NA	NA	NA	NA	NA	NA	NA	NA
**nEt-PFOSE**	NA	NA	9.30×10^{-5} (0.23)	NA	NA	NA	NA	NA	NA
**6:2 FTOH**	NA	NA	NA	NA	NA	NA	NA	NA	NA
**8:2 FTOH**	NA	NA	NA	NA	NA	NA	NA	NA	NA
**10:2 FTOH**	NA	NA	NA	NA	NA	8.47×10^{-5} (0.02)	NA	NA	NA

NA: not active up to the highest tested concentration (C_{max}).
/: not tested.

Table S13. Maximum concentration and cell responses of 16 PFAS in five cell-based bioassays. $EC_{10,nom}$ is the nominal effective concentration triggering 10% agonistic effects on human (h)PPARγ, rat (r)PPARγ, hPPARα, rPPARα and hERs. Data were from Evans et al.[10] and $EC_{10,nom}$ were re-evaluated using a linear concentration-response model to make the literature data comparable with the baseline toxicity prediction model. Specificity ratio (SR, Eq. 13) of 16 PFAS were calculated with baseline toxicity prediction models for anionic PFAS (Eq. 18) and neutral chemicals (Eq. 19) with the scenario of the generic cell.

**Abbreviation**	**C_{max} [mol/L]**	**hPPARγ $EC_{10,nom}$ (SR)**	**rPPARγ $EC_{10,nom}$ (SR)**	**hPPARα $EC_{10,nom}$ (SR)**	**rPPARα $EC_{10,nom}$ (SR)**	**hER $EC_{10,nom}$ (SR)**
**Anionic at pH=7.4 (11)**						
**PFHxA**	1.00×10^{-3}	7.82×10^{-4} (1.45)	8.38×10^{-4} (1.35)	2.04×10^{-4} (5.54)	NA	NA
**PFOA**	1.00×10^{-3}	2.78×10^{-4} (4.07)	5.11×10^{-4} (2.22)	3.89×10^{-5} (29.06)	NA	NA
**PFNA**	1.00×10^{-3}	8.91×10^{-4} (1.27)	NA	7.14×10^{-5} (15.84)	NA	NA
**PFDA**	1.00×10^{-3}	NA	NA	3.11×10^{-4} (3.64)	NA	NA
**PFMOAA**	1.00×10^{-3}	NA	NA	3.54×10^{-4} (3.20)	NA	NA
**HFPO-DA-AS**	1.00×10^{-3}	1.32×10^{-4} (8.54)	3.28×10^{-4} (3.45)	2.47×10^{-5} (45.84)	5.10×10^{-5} (22.21)	NA
**HFPO-DA**	1.00×10^{-3}	2.19×10^{-4} (5.17)	2.70×10^{-4} (4.19)	1.52×10^{-5} (74.64)	5.00×10^{-5} (22.65)	NA
**PFBS**	1.00×10^{-3}	4.28×10^{-4} (2.64)	6.73×10^{-4} (1.68)	2.86×10^{-4} (3.96)	NA	NA
**PFHxS**	1.00×10^{-3}	2.04×10^{-4} (5.53)	7.66×10^{-4} (1.48)	1.92×10^{-4} (5.89)	NA	4.31×10^{-4} (2.62)
**PFOS**	1.00×10^{-3}	8.36×10^{-4} (1.35)	NA	1.38×10^{-4} (8.21)	NA	NA
**NBP2**	1.00×10^{-3}	9.94×10^{-4} (1.14)	8.98×10^{-4} (1.26)	7.63×10^{-5} (14.84)	NA	NA
**Partially charged at pH=7.4 (1)**						
**PFOSA**	1.00×10^{-3}	NA	NA	NA	NA	NA
**Neutral at pH=7.4 (4)**						
**nEt-PFOSA**	1.00×10^{-3}	7.75×10^{-4} (1.46)	8.61×10^{-4} (1.32)	7.50×10^{-4} (1.51)	NA	NA
**4:2 FTOH**	1.00×10^{-3}	NA	NA	NA	NA	NA
**6:2 FTOH**	1.00×10^{-3}	NA	NA	NA	NA	2.19×10^{-4} (5.17)
**8:2 FTOH**	1.00×10^{-3}	NA	NA	NA	NA	NA

NA: not active up to the maximum concentration (C_{max}).

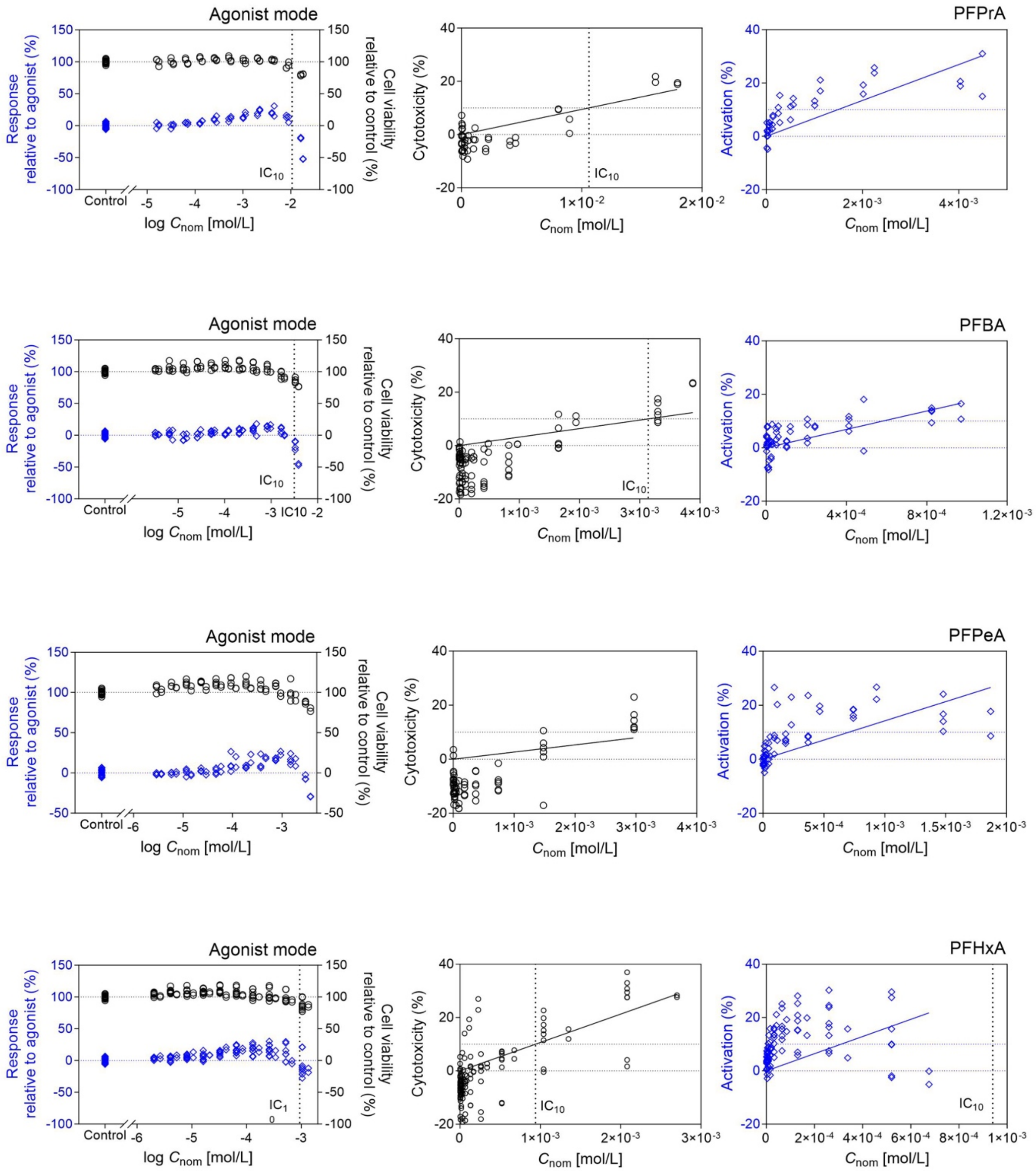

Figure S11. Agonistic mode and antagonistic mode of PPARγ-GeneBLAzer reporter gene assays of 24 PFAS.

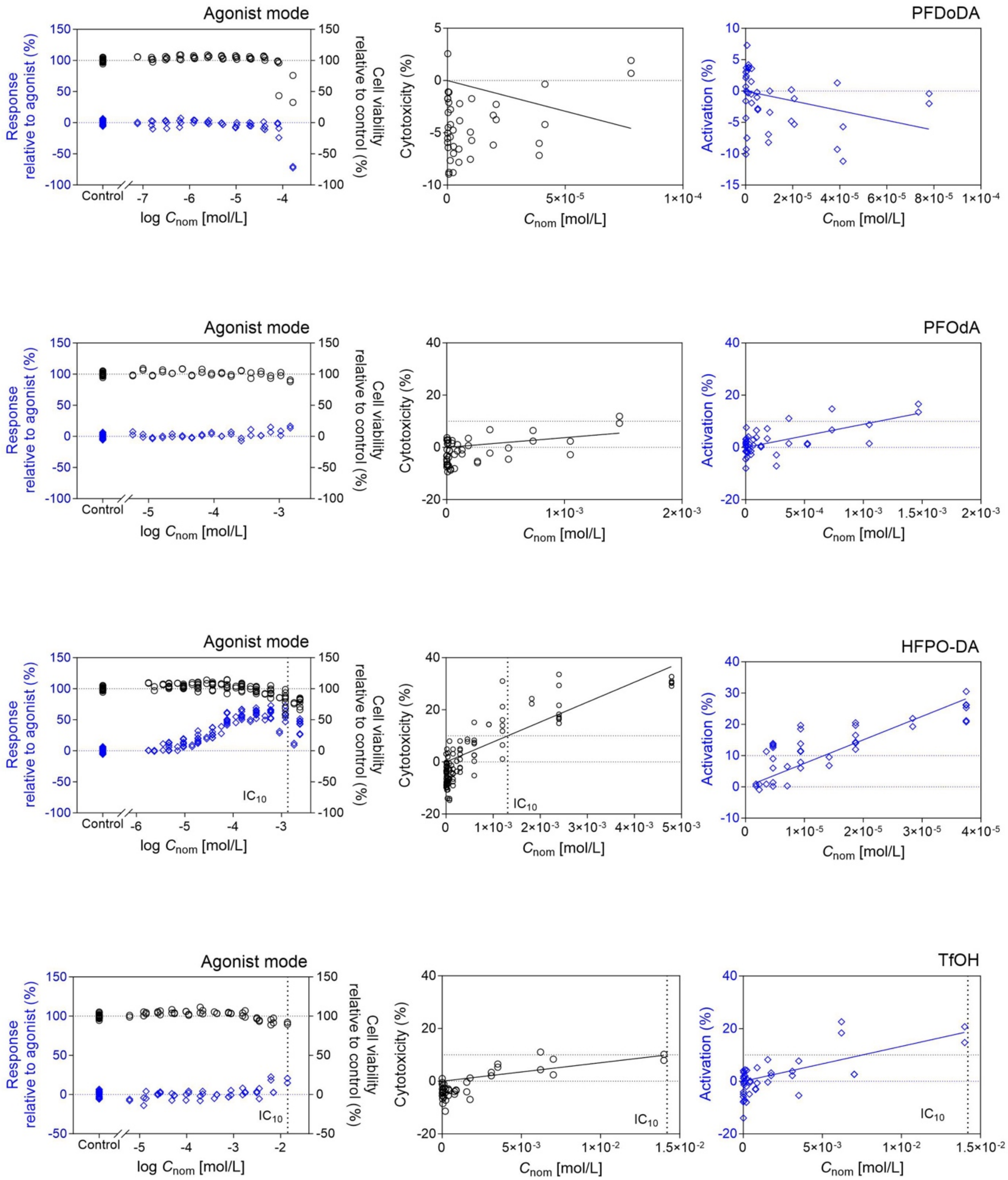

Figure S11 continued. Agonistic mode and antagonistic mode of PPARγ-GeneBLAzer reporter gene assays of 24 PFAS.

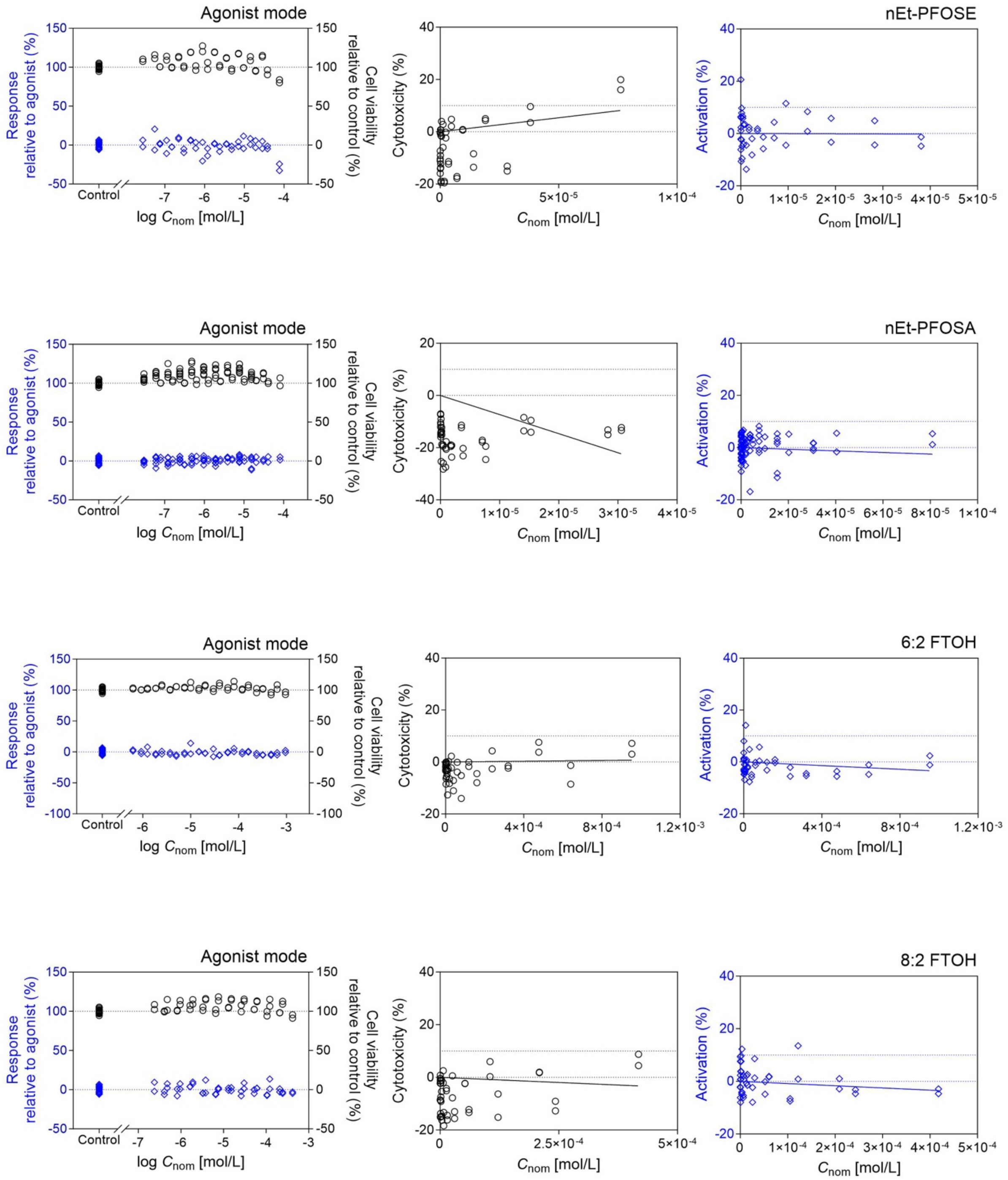

Figure S11 continued. Agonistic mode and antagonistic mode of PPARγ-GeneBLAzer reporter gene assays of 24 PFAS.

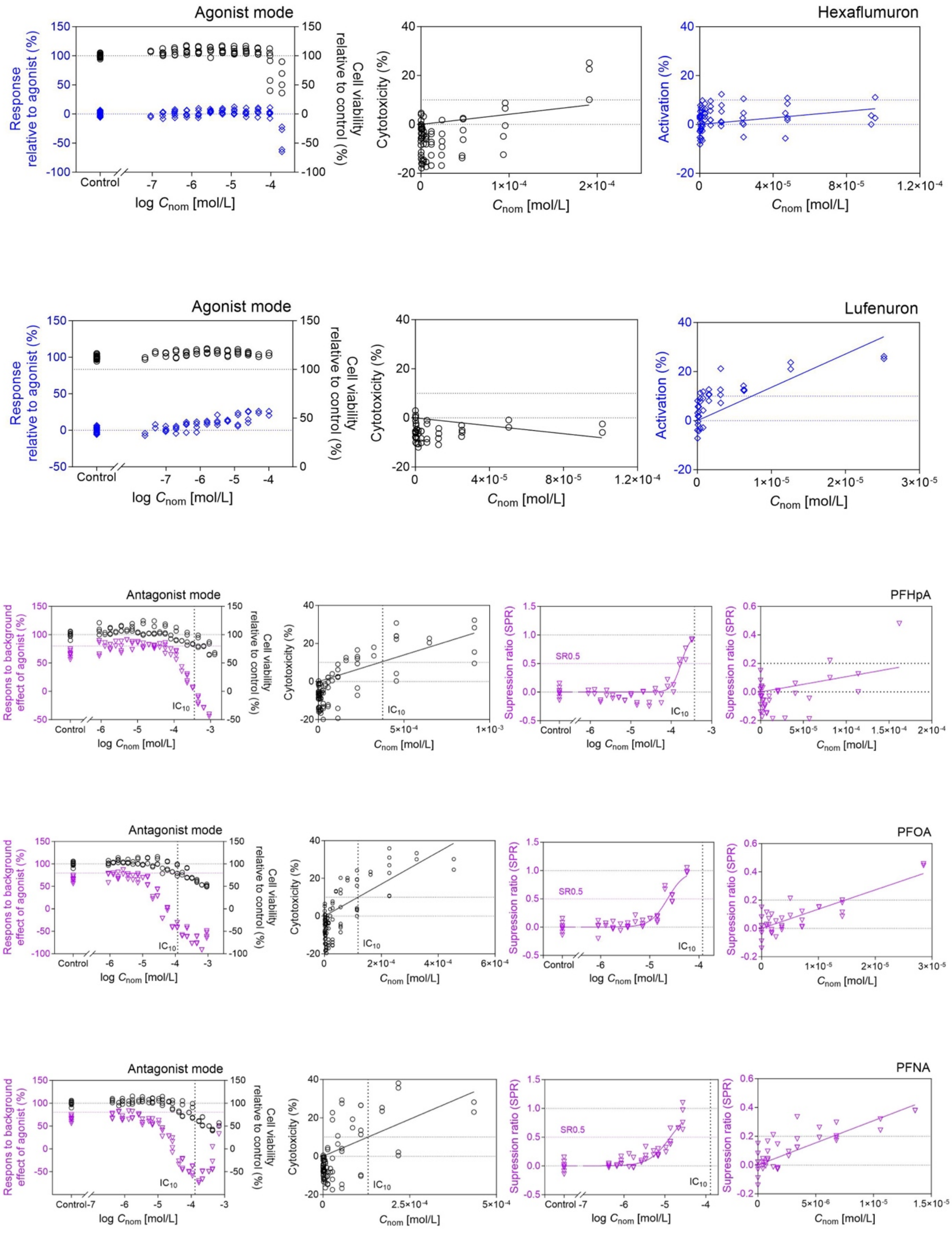

Figure S11 continued. Agonistic mode and antagonistic mode of PPARγ-GeneBLAzer reporter gene assays of 24 PFAS.

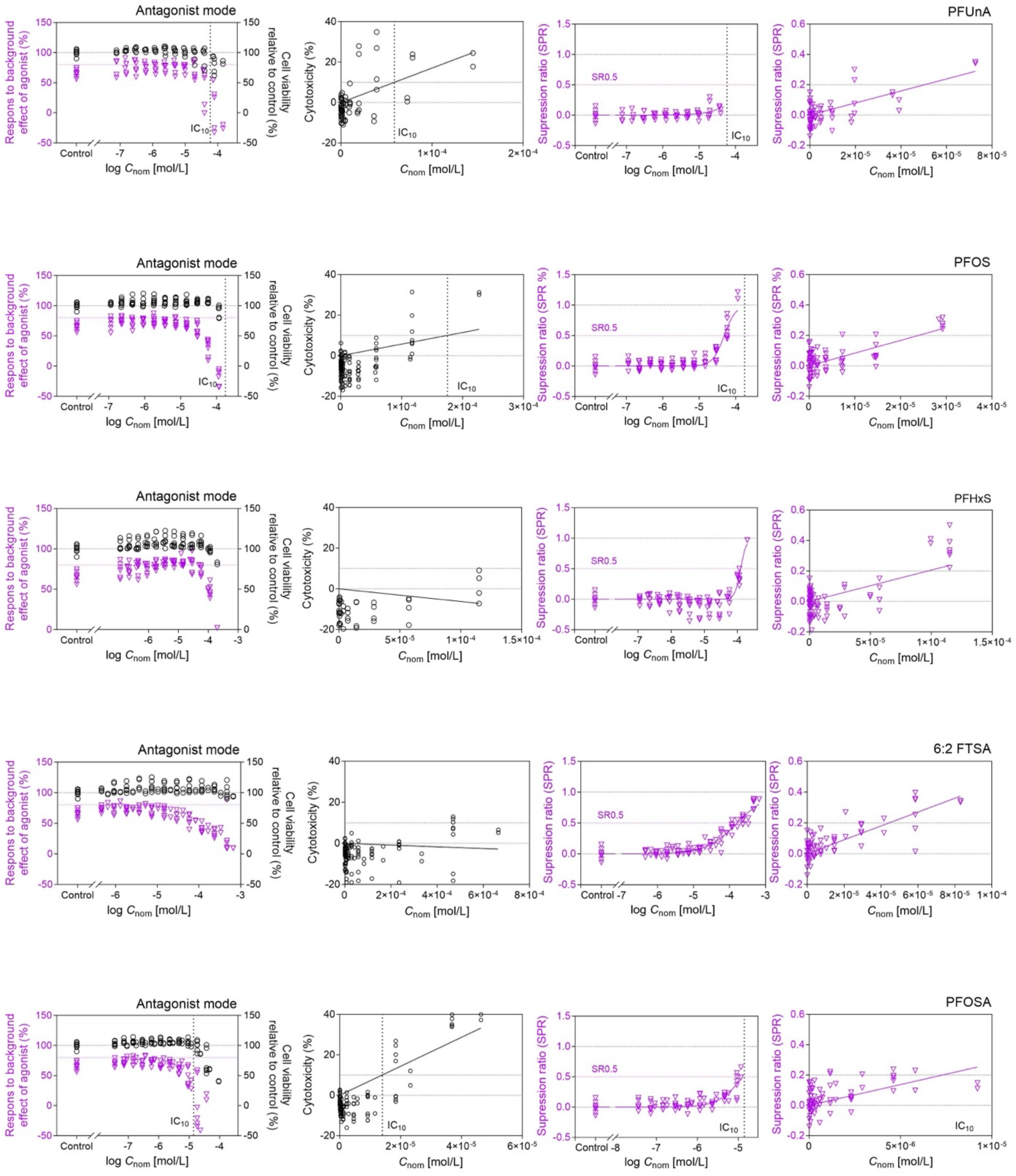

Figure S11 continued. Agonistic mode and antagonistic mode of PPARγ-GeneBLAzer reporter gene assays of 24 PFAS.

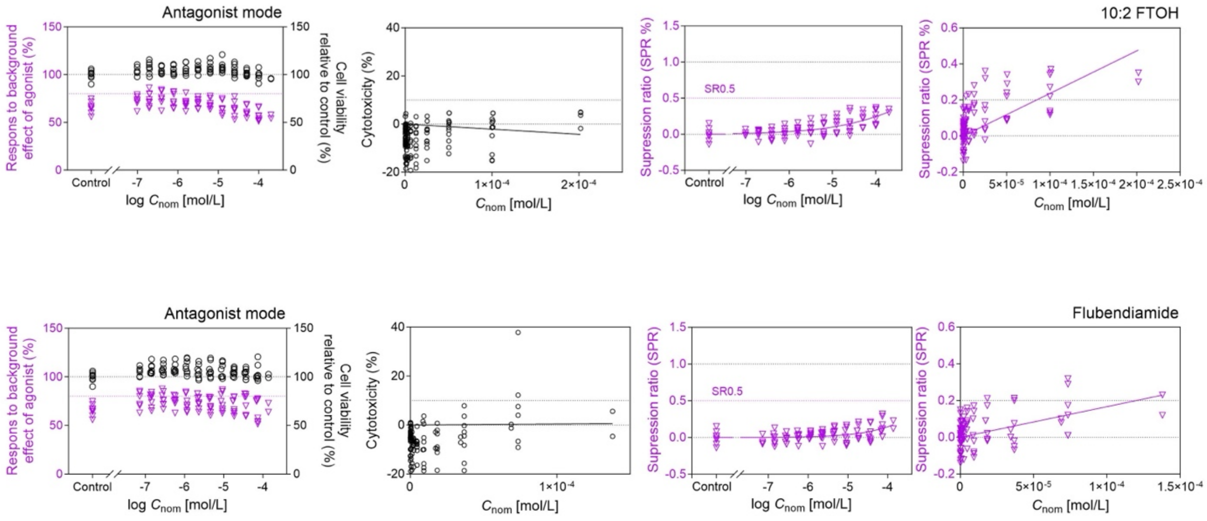

Figure S11 continued. Agonistic mode and antagonistic mode of PPARγ-GeneBLAzer reporter gene assays of 24 PFAS.

Reference

(1) Henneberger, L.; Muhlenbrink, M.; Konig, M.; Schlichting, R.; Fischer, F. C.; Escher, B. I. Quantification of freely dissolved effect concentrations in in vitro cell-based bioassays. *Arch Toxicol* **2019**, *93* (8), 2295-2305. DOI: 10.1007/s00204-019-02498-3.
(2) Qin, W.; Henneberger, L.; Huchthausen, J.; Konig, M.; Escher, B. I. Role of bioavailability and protein binding of four anionic perfluoroalkyl substances in cell-based bioassays for quantitative in vitro to in vivo extrapolations. *Environ Int* **2023**, *173*, 107857. DOI: 10.1016/j.envint.2023.107857.
(3) Escher, B. I.; Neale, P. A.; Villeneuve, D. L. The advantages of linear concentration-response curves for in vitro bioassays with environmental samples. *Environ Toxicol Chem* **2018**, *37* (9), 2273-2280. DOI: 10.1002/etc.4178.
(4) Escher, B. I.; Dutt, M.; Maylin, E.; Tang, J. Y.; Toze, S.; Wolf, C. R.; Lang, M. Water quality assessment using the AREc32 reporter gene assay indicative of the oxidative stress response pathway. *J Environ Monit* **2012**, *14* (11), 2877-2885. DOI: 10.1039/c2em30506b.
(5) Droge, S. T. J. Membrane-Water Partition Coefficients to Aid Risk Assessment of Perfluoroalkyl Anions and Alkyl Sulfates. *Environ Sci Technol* **2019**, *53* (2), 760-770. DOI: 10.1021/acs.est.8b05052.
(6) Ebert, A.; Allendorf, F.; Berger, U.; Goss, K. U.; Ulrich, N. Membrane/Water Partitioning and Permeabilities of Perfluoroalkyl Acids and Four of their Alternatives and the Effects on Toxicokinetic Behavior. *Environ Sci Technol* **2020**, *54* (8), 5051-5061. DOI: 10.1021/acs.est.0c00175.
(7) Endo, S.; Goss, K. U. Predicting partition coefficients of Polyfluorinated and organosilicon compounds using polyparameter linear free energy relationships (PP-LFERs). *Environ Sci Technol* **2014**, *48* (5), 2776-2784. DOI: 10.1021/es405091h.
(8) Endo, S.; Goss, K. U. Serum albumin binding of structurally diverse neutral organic compounds: data and models. *Chem Res Toxicol* **2011**, *24* (12), 2293-2301. DOI: 10.1021/tx200431b.
(9) Endo, S.; Bauerfeind, J.; Goss, K. U. Partitioning of neutral organic compounds to structural proteins. *Environ Sci Technol* **2012**, *46* (22), 12697-12703. DOI: 10.1021/es303379y.
(10) Evans, N.; Conley, J. M.; Cardon, M.; Hartig, P.; Medlock-Kakaley, E.; Gray, L. E., Jr. In vitro activity of a panel of per- and polyfluoroalkyl substances (PFAS), fatty acids, and pharmaceuticals in peroxisome proliferator-activated receptor (PPAR) alpha, PPAR gamma, and estrogen receptor assays. *Toxicol Appl Pharmacol* **2022**, *449*, 116136. DOI: 10.1016/j.taap.2022.116136.
